# The *C. elegans* H3K27 Demethylase UTX-1 Is Essential for Normal Development, Independent of Its Enzymatic Activity

**DOI:** 10.1371/journal.pgen.1002647

**Published:** 2012-05-03

**Authors:** Julien Vandamme, Gaëlle Lettier, Simone Sidoli, Elia Di Schiavi, Ole Nørregaard Jensen, Anna Elisabetta Salcini

**Affiliations:** 1 Biotech Research and Innovation Centre (BRIC), University of Copenhagen, Copenhagen, Denmark; 2 Centre for Epigenetics, University of Copenhagen, Copenhagen, Denmark; 3 Centre for Epigenetics, Department of Biochemistry and Molecular Biology, University of Southern Denmark, Odense, Denmark; 4 Institute of Genetics and Biophysics “Adriano Buzzati Traverso,” Consiglio Nazionale delle Ricerche (CNR), Naples, Italy; University of California San Diego, United States of America

## Abstract

Epigenetic modifications influence gene expression and provide a unique mechanism for fine-tuning cellular differentiation and development in multicellular organisms. Here we report on the biological functions of UTX-1, the *Caenorhabditis elegans* homologue of mammalian UTX, a histone demethylase specific for H3K27me2/3. We demonstrate that *utx-1* is an essential gene that is required for correct embryonic and postembryonic development. Consistent with its homology to UTX, UTX-1 regulates global levels of H3K27me2/3 in *C. elegans*. Surprisingly, we found that the catalytic activity is not required for the developmental function of this protein. Biochemical analysis identified UTX-1 as a component of a complex that includes SET-16(MLL), and genetic analysis indicates that the defects associated with loss of UTX-1 are likely mediated by compromised SET-16/UTX-1 complex activity. Taken together, these results demonstrate that UTX-1 is required for many aspects of nematode development; but, unexpectedly, this function is independent of its enzymatic activity.

## Author Summary

Chromatin organization influences gene expression, and its regulation is crucial to achieve correct cellular differentiation and development in multicellular organisms. Histone demethylases are among several factors responsible for regulating chromatin dynamics. Here we report on the biological functions of UTX-1, the *C. elegans* homologue of the mammalian histone demethylase UTX, which specifically catalyzes the demethylation of di- and tri-methylated lysine 27 of histone H3 (H3K27me2/3). Indeed, we demonstrate that UTX-1 regulates global levels of H3K27me2/3 in *C. elegans*, a mark generally associated with silencing of gene expression. We also show that *utx-1* is an essential gene that is required for correct embryonic and postembryonic development. Specifically, the loss of *utx-1* results in developmental defects, sterility, and embryonic lethality. Surprisingly, our data show that the catalytic activity of UTX-1 is not required for its developmental functions. Our biochemical and genetic analyses indicate that loss of UTX-1 compromises the activity of the SET-16(MLL) complex, which UTX-1 is an integral part of. Taken together, these results demonstrate that UTX-1 plays an essential role in development independent of its enzymatic activity.

## Introduction

The proper development of multicellular organisms requires strict regulation of cell-specific gene expression to ensure appropriate cell fate specification, cellular differentiation, and organogenesis. In addition to transcription factors, gene expression is controlled by chromatin organization, which is regulated by chromatin-remodelling factors and the post-translational modifications of histone proteins [1]–[3].

An important post-translational modification is the mono- (me), di- (me2), or tri- (me3) methylation of lysine residues (K) on the tail of histone 3 (H3). Specifically, the methylation of specific lysine residues plays a major role in the maintenance of active and silent gene expression states. The combination of H3 K4, K36, and K79 tri-methylation generally marks transcriptionally active regions, whereas H3 K9 and K27 tri-methylation marks regions of transcriptionally silenced genes [2]. The levels of methylation are modulated by the action of histone methyltransferases (HMTs) and histone demethylases (HDMs). The largest group of histone demethylases contains a Jumonji C-domain (JmjC) that catalyzes the demethylation of specific lysine and arginine residues by an oxidative reaction requiring iron [Fe(II)] and α-ketoglutarate (αKG) as cofactors [4]. There are 28 JmjC-containing proteins in humans, grouped in different families, and the majority of these are evolutionarily conserved [5]. The KDM6 subfamily (UTX/UTY/JMJD3) was shown to catalyze the demethylation of H3K27me2/3 [6]–[11], and the individual members were shown to regulate differentiation in several cellular systems [6], [7], [10]. In *C. elegans*, there are four KDM6 family members: *jmjd-3.1*, *jmjd-3.2, jmjd-3.3*, closely related to JMJD3, and *utx-1*, the unique homologue of the human UTX/UTY. The functional role of these proteins in nematodes is not well defined. *jmjd-3.1* has been reported to regulate somatic gonadal development [6], while *utx-1* has been implicated in vulva differentiation and aging [12]–[14].

In this report, we have analyzed the developmental functions of UTX-1. We show that *utx-1* plays a vital role during embryogenesis and acts in several aspects of nematode postembryonic development. Surprisingly, we found that the catalytic activity of UTX-1 is not of critical importance for UTX-1 function in development. Genetic and biochemical analyses indicate that UTX-1 acts through a SET-16(MLL)/UTX-1 complex and that the primary role of UTX-1 resides in the regulation of the activity of this complex.

## Results

### Loss of *utx-1* results in reduced fertility and lethality

**Figure 1:**
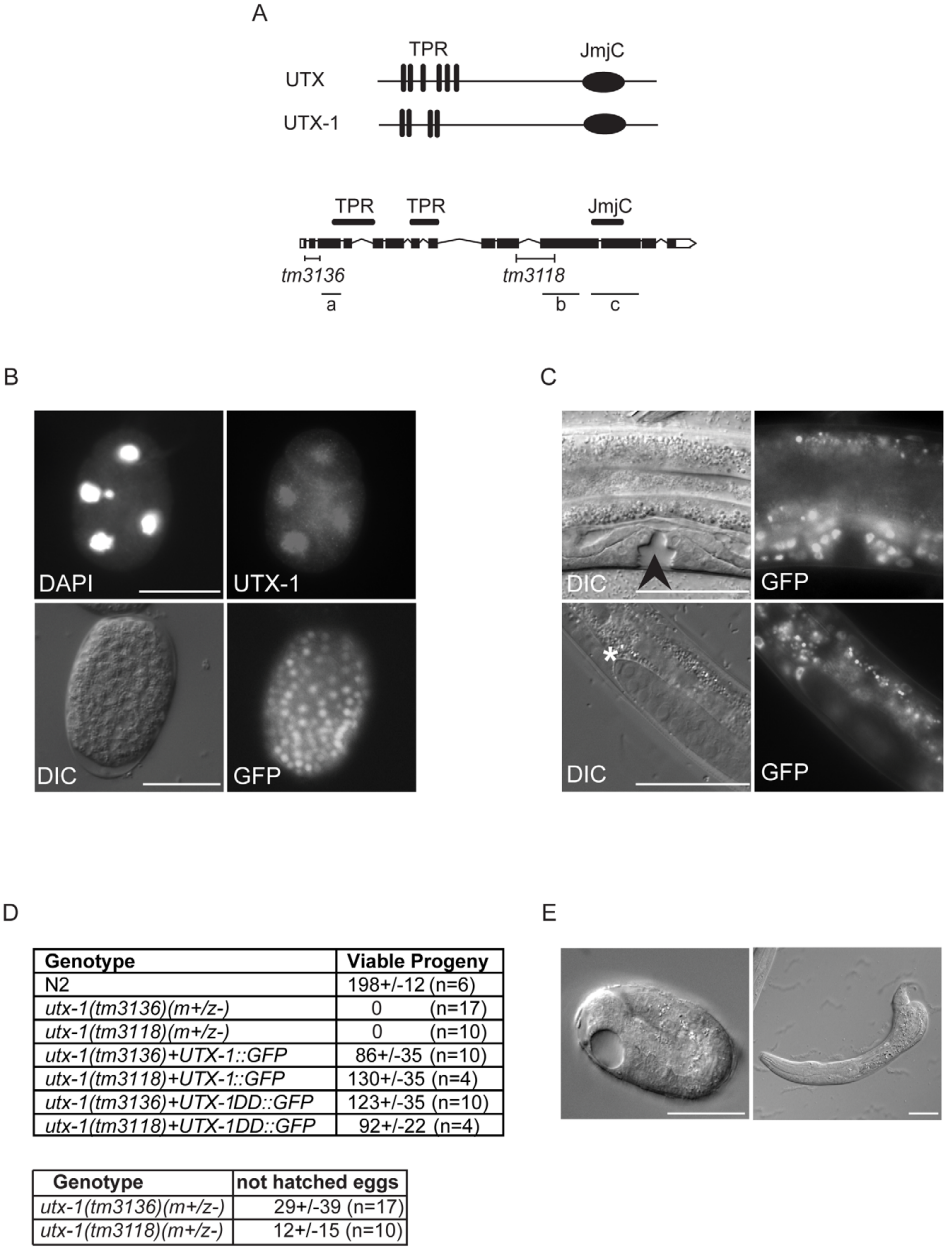
UTX-1 expression and *utx-1* embryonic phenotypes. (A) Top: Human UTX and the *C. elegans* homologue UTX-1. TPRs, tetratricopeptide repeats; JmjC, Jumonji C domain. Bottom: Genomic organization of *utx-1*. Black H-shaped lines indicate the position of the *tm3136* and *tm3118* deletions. Black lines indicate the position of the different *RNAi* fragments (a, b, and c) used in this study. (B) UTX-1 expression during embryogenesis analyzed by immunostaining with an anti-UTX-1 antibody (top panel, right) and by epifluorescence (bottom panel, right). DAPI staining and Nomarski (DIC) images are also shown on the left. (C) UTX-1 expression by epifluorescence (right panels) during larval development. Nomarski (DIC) images are shown on the left panels. Asterisks indicate the distal tip cell, arrow head the forming vulva. Animals are oriented anterior to the left. (D) Brood size of wild type, *utx-1* mutant worms and rescued *utx-1* lines. Progeny is given as the average number of viable progeny per worm ± SD. The number of laid, not hatched, eggs counted in *utx-1 (m+/z−)* mutants is reported in the lower table. *utx-1+UTX-1::GFP* and *utx-1+UTX-1DD::GFP*, are *utx-1* transgenic lines expressing wild-type or catalytically inactive mutant of UTX-1, respectively, as extrachromosomal arrays. (E) Representative Nomarski (DIC) images of a *utx-1(tm 3136)* mutant embryo and escaper L1 larva. Similar phenotypes are observed in *utx-1(tm 3118)* (not shown). Bars in B and E are 20 µm, in C 50 µm. Animals are oriented anterior to the left.

*C. elegans D2021.1* encodes for a predicted protein of 134 kDa that has high homology and co-linearity with the mammalian UTX/UTY proteins (Figure 1A); thus we named this gene and its product *utx-1* and UTX-1, respectively. UTX-1 is expressed in most, if not all, nuclei of early and late stage embryos (Figure 1B) as well as during all of the larval stages and into adulthood (Figure 1C), suggesting that UTX-1 could have a functional role throughout *C. elegans* development. To determine the biological function of UTX-1 two deletion mutant strains, *utx-1(tm3136)* and *utx-1(tm3118)*, were analyzed (Figure 1A). The *tm3136* allele is a 236 bp deletion that creates a premature stop codon, potentially encoding a truncated protein of only 28 amino acids, and very likely producing a null mutant. The *tm3118* allele is an out–of-frame deletion of 547 bp. The truncated protein potentially retains the first 620 amino acids, but is lacking the JmjC domain and catalytic activity. The two alleles have similar phenotypes suggesting that they are both loss of function mutants.

**Figure 2:**
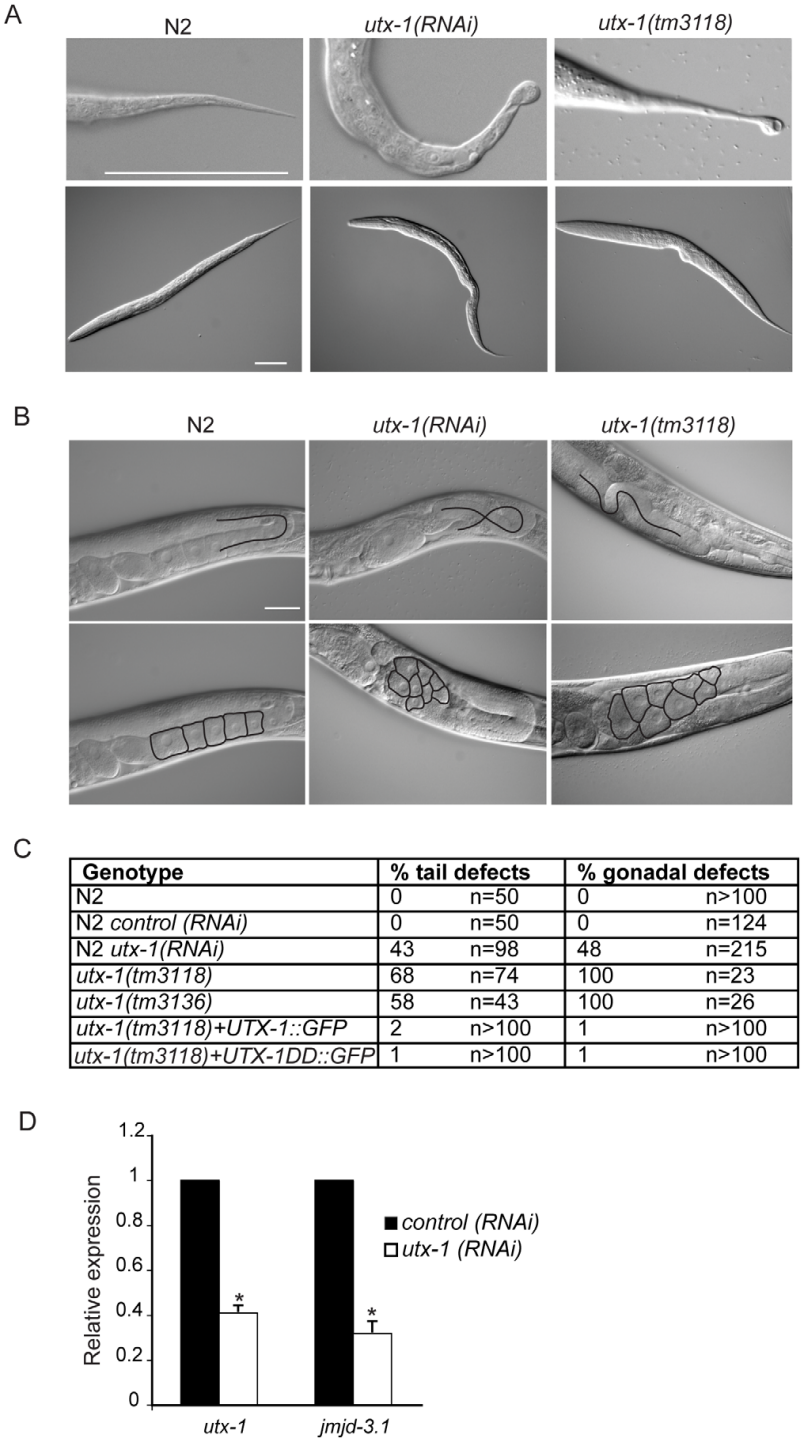
*utx-1* postembryonic phenotypes. (A) Representative DIC images of L1 larvae tails (upper panel) and L1 larvae (lower panel) of N2, *utx-1(RNAi)* and *utx-1(tm3118)* animals. Animals are oriented anterior to the left. Scale bar is 50 µm. (B) Representative DIC images of gonads in adults N2, *utx-1(RNAi)* and *utx-1(tm3118)* animals. Scale bar is 25 µm. In the upper panels, blacks lines indicate the migration of the gonad arm. In the lower panels, black lines indicate the contours of the oocytes. Animals are oriented anterior to the left. (C) Percentages of posterior (% tail defects) and gonad (% gonadal defects) defects in the indicated strains are shown. For *RNAi*, F1 larvae and adults from at least three independent experiments were analyzed. (D) *utx-1* and *jmjd-3.1* mRNA levels in embryos after control (black bars) or *utx-1* (white bars) *RNAi* treatment as measured by qPCR, using *rpl-26* mRNA as internal control. *P<0.01 (Student's t-test).

Homozygous *utx-1* mutant worms that are derived from heterozygous mothers providing maternal UTX-1, *utx-1(m+/z−)*, are viable and reach adulthood. However, they produce only a few, mostly unviable, *utx-1(m−/z−)* eggs (Figure 1D and 1E), suggesting that UTX-1 is required for embryogenesis and that the lack of UTX-1 can be overcome by maternal contribution. Analysis of the dead embryos revealed that mutant *utx-1* animals mainly arrested as late embryos (Figure 1E). Dead L1 larvae, with misshapen bodies (Figure 1E), were rarely observed (5%, n>200). A p*utx-1::UTX-1::GFP* (*UTX-1::GFP*) translational reporter as extra-chromosomal array was able to rescue the embryonic lethal phenotype observed in heterozygous *utx-1(m−/z−)* progeny from mothers carrying either the *tm3136* or *tm3118* allele (Figure 1D) in several transgenic lines, leading us to conclude that UTX-1 is essential for embryogenesis and that the zygotic expression of UTX-1 is sufficient to restore embryonic viability. Indeed, progeny that did not receive the transgene from the mother, died as late stage embryos (not shown) or malformed L1 larvae (Figure S1A), suggesting that UTX-1 is not required for very early embryogenesis. In agreement with this, analysis of epithelial junctions using an *AJM-1::*GFP translational reporter [15] suggests that the morphology of *utx-1(m−/z−)* embryos, that did not inherit the transgene, was normal at early stages and progressively deteriorated throughout development (Figure S1B). Analysis of markers for intestinal (*elt-2::GFP*), muscular (*hlh-1::GFP* and *myo-2::GFP*), and hypodermal (*dpy-7::GFP*) cells revealed that these cell lineages are correctly established in *utx-1(tm3118)* mutant worms (Figures S2, S3, S4, S5). However, a progressive loss of *myo-3::GFP* transgene expression during embryogenesis was observed and little GFP signal was detected in L1 escapers (Figure S6), suggesting that defects in muscle function might account, at least in part, for the lethality of *utx-1* null animals (Figure 2B).

### Reduction of *utx-1* results in posterior defects and in aberrant gonad migration and organization

To determine the function of UTX-1 at later developmental stages, we analyzed both the *utx-1(m+/z−)* mutant worms, in which the zygotic contribution of UTX-1 is lost, and wild-type worms in which *utx-1* expression was downregulated by feeding RNA interference (*RNAi*). RNA interference, with constructs targeted to three different regions of *utx-1* (Figure 1A), resulted in an approximately 60% reduction of *utx-1* mRNA in F1 progeny and in a significant reduction of UTX-1 protein expression (Figure S7A). In agreement with the phenotype of *utx-1(m+/z−)* mutant animals, the *utx-1(RNAi)* F1 animals had reduced fertility (Figure S7B). Furthermore, variable defects, often located posteriorly, were observed in about 40% of the *utx-1(RNAi)* worms (Figure 2A and 2C). The posterior defects observed in animals treated with *utx-1(RNAi)* were very similar to the defects observed in *utx-1(m−/z−)* dead larvae (Figure 2A, 2C and Figure S8). This demonstrates that RNA interference can be used to efficiently analyze the postembryonic roles of UTX-1, and that the posterior phenotype in *utx-1(m−/z−)* is due to the loss of *utx-1*. Importantly, transgenic expression of wild-type *utx-1* fully rescued the posterior defects in larvae.

The reduced fertility observed in *utx-1(m+/z−)* animals might be due to a regulatory role for UTX-1 in either somatic gonad or germline development. Homozygous mutant *utx-1(m+/z−)* animals from heterozygous animals generally develop germlines with correct proliferation and differentiation patterns (not shown), as demonstrated by the fact that oocytes are formed (Figure 2B) and by an ability to lay a few dead embryos, suggesting that the sterility is not related to a germline defect. However, animals lacking UTX-1 activity have defects in gonad migration and oocyte organization. The shape of the gonad is dictated by the coordinated migration of two distal tip cells (DTCs), which are part of the somatic gonad structure and move away from the gonad primordium during postembryonic development, leading to two consecutive turns forming the U-shaped gonad arms observed in adult animals. Using transgenic animals carrying a distal tip cell marker, *lag-2::GFP* [16], we observed aberrant gonadal migration in 42% (n = 176) of the *utx-1(RNAi)* animals. Morphological analysis by DIC of *utx-1(RNAi)* animals further confirmed that 48% (n = 215) of the animals showed a failure to turn or abnormal turning of at least one gonad arm (Figure 2B and 2C), and these animals often (41%, n = 137) developed misshapen gonads, with an enlargement of the proximal end of the gonad arms and a misorganization of oocytes (Figure 2B). These gonad phenotypes were also identified in *utx-1(m+/z−)* mutant animals (Figure 2B, 2C and Figure S9), and they were efficiently rescued by the *UTX-1::GFP* transgene, reinforcing that these aberrations are caused by the loss of *utx-1* (Figure 2C). The fact that the transgenic expression of wild-type *utx-1* is able to rescue the sterility and the gonadal phenotypes suggests that *utx-1* has a role in the somatic gonad rather than in the germline, where transgenes are normally silenced. Consistent with this, GFP-tagged UTX-1 is expressed in the distal tip cells during migration (Figure 1C) and other tissues of the somatic gonad, such as the sheath cells and the spermatheca (not shown) and not in the germline.

The aberrant migration and oocyte organization defects are similar those we reported for *jmjd-3.1* loss-of-function mutants, which encodes one of the *C. elegans* homologues of the JMJD3 family [6]. To determine if there is a link between these two observations, we tested if UTX-1 affected the expression of *jmjd-3.1* by performing quantitative PCR on *utx-1(RNAi)* animals. As shown in Figure 2D, *utx-1*(*RNAi*) animals have reduced levels of *jmjd-3.1*, suggesting that UTX-1 may, directly or indirectly, regulate *jmjd-3.1* expression. Additionally, an enhancement of the phenotype was not observed when *utx-1* was reduced in a *jmjd-3.1* mutant genetic background (see below), suggesting that both genes are acting in the same genetic pathway to regulate somatic gonadal development.

### UTX-1 demethylates H3K27me2/3, but the catalytic activity is not required for proper development

**Figure 3:**
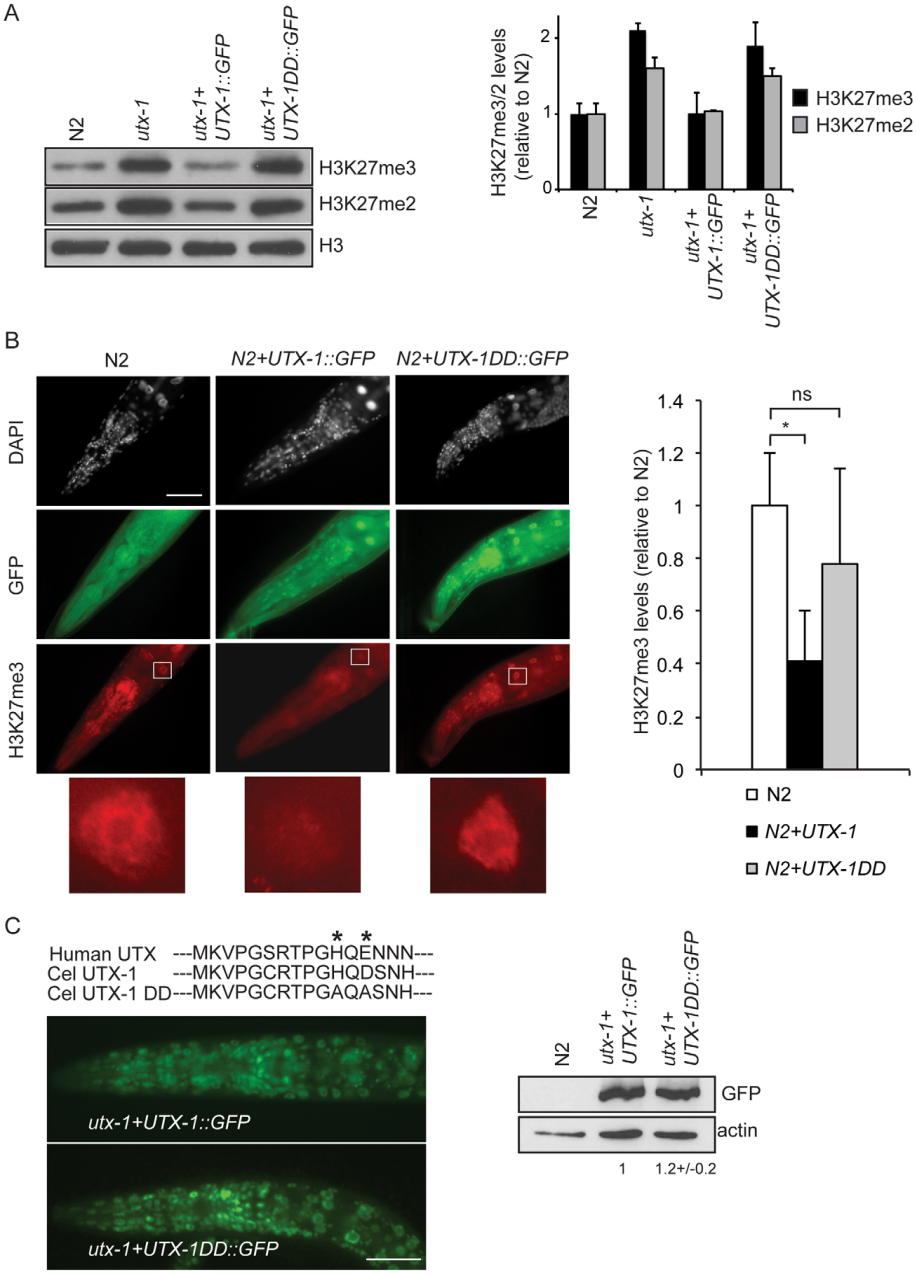
UTX-1 is an H3K27me2/3 demethylase. (A) Protein lysates from embryos of the indicated strains were probed with antibodies against H3K27me3 and H3K27me2. H3 was used as loading control. Quantification of the western blot is shown in the graphic on the right. Error bars indicate the standard deviation calculated using at least 2 replicates. The signals were quantified using ImageJ software and normalized to H3. The values are relative to N2 levels. Similar results were obtained with at least two different transgenic lines and in the two *utx-1* genetic backgrounds (*tm3118 and tm3136*). (B) Representative images of N2 expressing a translational construct for wild-type *(N2+UTX-1::GFP)* and catalytically inactive UTX-1 *(N2+UTX-1DD::GFP)* GFP fusion and GFP-negative siblings, fixed and stained with H3K27me3 antibody and DAPI. The white square encloses an intestinal cell, used for the H3K27me3 quantification. Enlargement of the white square is shown at the bottom of the panel. Quantification of H3K27me3 levels is shown in the graphic on the right. At least 25 cells for each genotype were quantified as described in Materials and Methods. Mean fluorescence + s.e.m. values of two independent experiments are displayed. *P<0.01. (Student's t-test). Animals are oriented anterior to the left. (C) Top: Alignment of a part of the Jumonji C domain of human UTX with UTX-1 and with the catalytically inactive UTX-1DD (DD = Demethylase Dead). Asterisks indicate two of the three conserved amino acids in the iron-binding domain (HXD/EX_n_H) of the JmjC-domain, modified in the UTX-1DD. Bottom: Epifluorescence of *utx-1* mutant animals, carrying a translational GFP fusion of wild-type UTX-1 (*utx-1+UTX-1::GFP*) or catalytically inactive UTX-1 (*utx-1+UTX-1DD::GFP*). Anterior parts of the animals are shown, with anterior to the left. On the right, lysates from L1 carrying the two transgenes were probed with GFP antibody. Actin was used as loading control. The signal was quantified using ImageJ program and normalized to actin.

UTX-1 belongs to the KDM6 family, of which members have been shown to catalyze the demethylation of H3K27me3 and H3K27me2 [17]. Several observations indicate that this role is conserved throughout the *C. elegans* life cycle. First, loss of the zygotic and maternal contributions of *utx-1* results in increased global levels of H3K27me2/3 at the embryonic stage (Figure 3A). Second, reduction of UTX-1 by RNA interference results in a significant increase of H3K27me3 levels at different larval stages (data not shown, [12], [18]). Third, exogenous expression of wild-type UTX-1 in *utx-1* null animals restores H3K27me3 to wild-type level (Figure 3A). Fourth, over-expression of UTX-1 in wild-type animals results in a significant reduction of global H3K27me3 levels (Figure 3B). Finally, the decreased level of H3K27me3 observed in N2 worms overexpressing UTX-1 (Figure 3B) is well correlated with the degree of *UTX-1::GFP* overexpression, as shown in Figure S10.

Next, we tested if the catalytic activity of UTX-1 is responsible for the phenotype observed in *utx-1* null mutants and *utx-1(RNAi)* animals. To this end, we expressed in *utx-1* mutants a GFP-tagged mutated form of the UTX-1 protein (for simplicity called *UTX-1DD::GFP, DD = Demethylase Dead*), carrying mutations in two of the three conserved amino acids in the iron-binding motif (HXD/EX_n_H) of the JmjC-domain (indicated by asterisks in Figure 3C). Several reports have shown that these amino acids are required for iron binding and thus for the catalytic activity of all JmjC-domain containing demethylases characterized so far [6]–[11], [19]–[21]. All *UTX-1::DD::GFP* transgenic lines generated (8/8) showed expression at levels similar to wild-type UTX-1 (Figure 3C) and were fertile and able to produce viable progeny (Figure 1D). Importantly, re-expression of catalytically inactive UTX-1 did not restore the wild-type level of H3K27me3 in *utx-1* null animals (Figure 3A) and did not influence the H3K27me3 level when overexpressed in wild-type animals (Figure 3B), thus confirming that the amino acids substitutions affected UTX-1 enzymatic activity. This unexpected result strongly indicates that the demethylase activity of UTX-1 is not important for either embryonic development or animal viability. Subsequently, we tested if the other observable phenotypes were dependent on UTX-1 enzymatic activity. Tail and gonadal defects were also efficiently rescued (Figure 2C) in 50% (4/8) of the transgenic lines, indicating that UTX-1, but not its catalytic activity, is required for correct posterior and gonadal development.

### *C. elegans* KDM6 family members do not compensate for the reduced H3K27me2/3 demethylase activity in *utx-1* mutant worms

**Figure 4:**
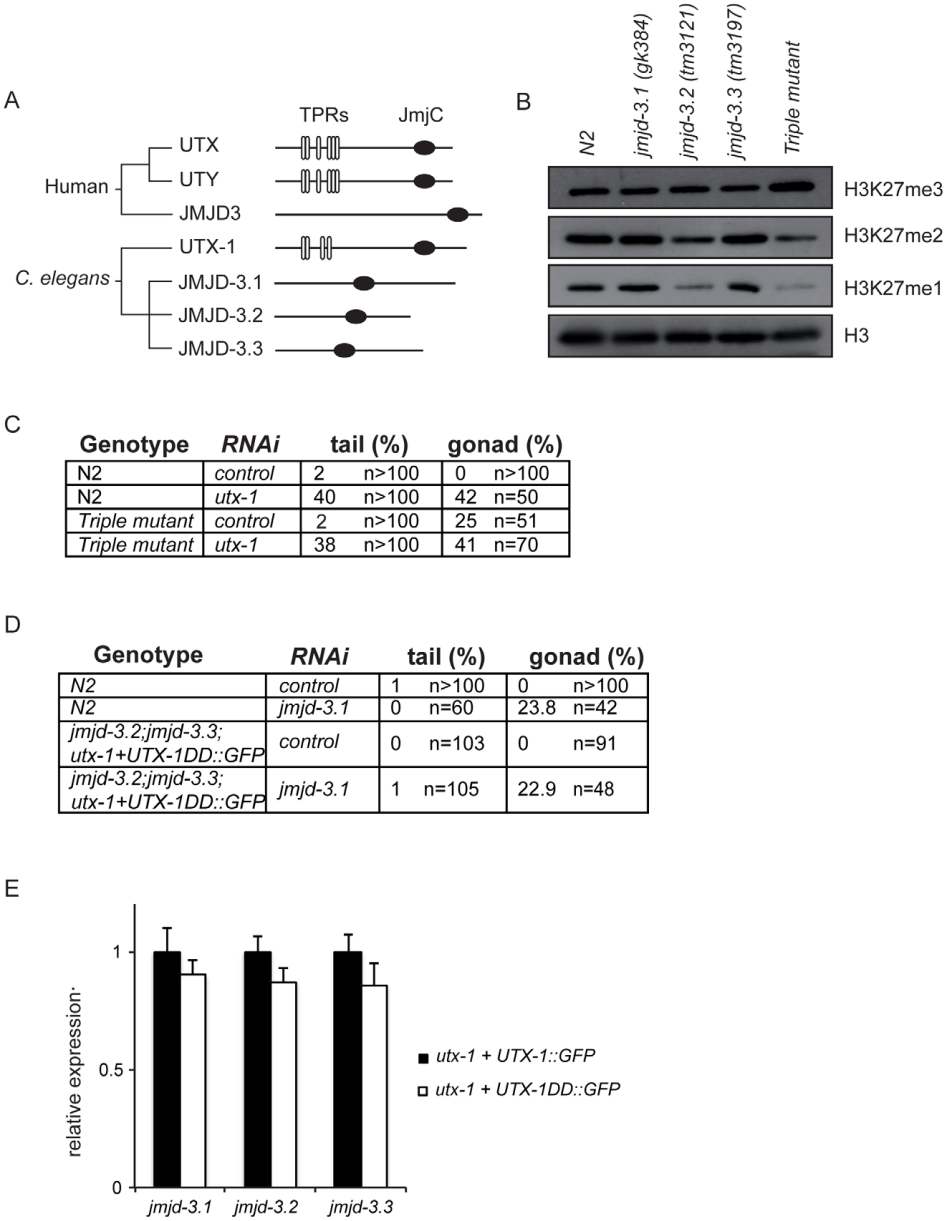
KDM6 family in *C. elegans*. (A) Phylogenetic cluster of human UTX, UTY, JMJD3 and homologous proteins in *C. elegans*. TPRs, tetratricopeptide repeats; JmjC, Jumonji C domain. (B) Protein lysates from eggs of the indicated strains were analyzed by western blot using the indicated antibodies. H3 was used as loading control. Quantification of the western blot is shown in Figure S11. (C) Percentage of tail and gonad defects observed in N2 and the triple mutant (*jmjd-3.1;jmjd-3.2;jmjd-3.3*) after treatment with control or *utx-1(RNAi)*. F1 animals from at least two independent experiments were scored. (D) Percentage of tail and gonad defects observed in N2 and the triple mutant (*jmjd-3.2; jmjd-3.3;utx-1+UTX-1DD::GFP*) after treatment with control or *jmjd-3.1(RNAi)*. F1 animals from at least two independent experiments were scored. (E) Levels of expression of the JMJD3-like genes in eggs derived from the *utx-1(tm3136)* mutant strain rescued with the catalytically inactive *utx-1 (utx-1+UTX-1DD::GFP)* relative to *utx-1(tm3136)* rescued with *utx-1* wild-type *(utx-1+UTX-1::GFP)*. *rpl-26* mRNA was used as internal control for normalization.

*jmjd-3.1*, *jmjd-3.2*, and *jmjd-3.3* (Figure 4A) are *C. elegans* KDM6 family members closely related to human JMJD3. Animals carrying mutations in one of these genes are viable, fertile (not shown), and do not show up-regulated levels of H3K27me3 by western blot analysis (Figure 4B and Figure S11B). However, triple mutant worms carrying deletions in all three JMJD3-like genes showed increased global levels of H3K27me3 (Figure 4B and Figure S11B), suggesting that these proteins are H3K27me3 demethylases and might act redundantly with UTX-1. Several lines of evidences indicate that the JMJD3-like genes do not function redundantly with UTX-1. Analysis of the transcriptional expression levels of the JMJD3-like genes in wild-type worms indicated that only *jmjd-3.1* is expressed at levels comparable to *utx-1*, while *jmjd-3.2* and *jmjd-3.3* are only weakly expressed, in particular during larval stages (Figure S11A). Furthermore, the transcriptional expression pattern of the JMJD3-like genes appeared generally restricted to specific tissues or, as in the case for *jmjd-3.2*, even to few cells (Figure S11C); this is in contrast to the broad expression pattern of UTX-1. In addition, the triple mutant lacking the JMJD3-like genes is viable and fertile, with no defects in the posterior region of the body (Figure 4C) and with only minor gonadal defects (Figure 4C), most likely due to the absence of *jmjd-3.1*. Importantly, the down-regulation of *utx-1* by RNA interference in the triple mutant genetic background did not exacerbate the posterior or the gonadal defects associated with *utx-1* reduction in wild-type animals (Figure 4C). Taken together, these results imply that the members of the KDM6 class do not act redundantly.

However, in light of the unexpected results obtained with the catalytically inactive UTX-1 mutant, it is important to take into consideration the possibility that JMJD3-like genes could, nevertheless, compensate for the lack of UTX-1 activity in *utx-1* mutant worms expressing the catalytically inactive form of UTX-1. In this case, we would expect that the loss or reduction of other H3K27me3 demethylases in the *utx-1* mutant rescued with the catalytically inactive UTX-1 would result in *utx-1*-specific abnormalities (posterior defects and aberrant gonadal migration). To test this hypothesis, we generated a triple mutant *jmjd-3.2; jmjd-3.3;utx-1+UTX-1DD::GFP* in which the fourth member of the KDM6 family, *jmjd-3.1*, was down-regulated by RNA interference. In this genetic background, no posterior defects were observed and the degree of gonadal defects was similar to those observed in wild-type animals under the same conditions (Figure 4D). Furthermore, quantitative PCR showed no increased expression levels of the JMJD3-like genes in the rescued transgenic line *utx-1+UTX-1DD::GFP* compared to *utx-1+UTX-1::GFP* (Figure 4E). These results, together with the fact H3K27me3 levels are still up-regulated in *utx-1* expressing the catalytically inactive form of UTX-1 (Figure 3A), strongly indicate that the JMJD3-like proteins do not compensate for the lack of UTX-1 catalytic activity and that the catalytic activity of UTX-1 is not required for proper development.

### UTX-1 acts in the SET-16/MLL complex

**Figure 5:**
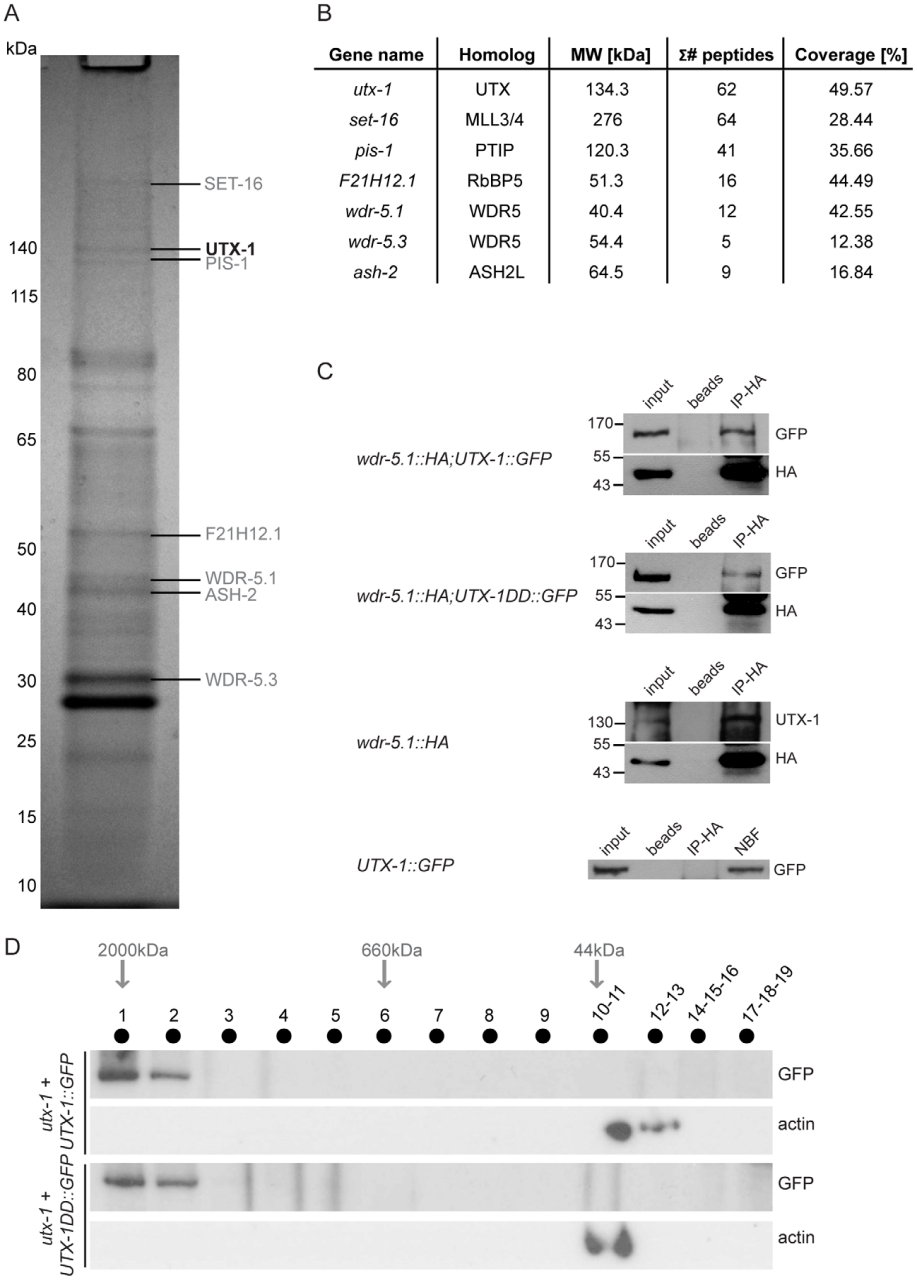
UTX-1 is part of a MLL-like complex. (A) Immunoprecipitation of GFP tagged UTX-1 from a mixed population (eggs and adults) of *utx-1(tm3118)* rescued with UTX-1::GFP. Affinity purified proteins were resolved by SDS-PAGE and stained with colloidal Coomassie. Homologues of the mammalian UTX-MLL complex co-eluted with the bait and identified by LC-MS/MS are listed in grey. Position of the bait protein is shown in black. Molecular weight markers are indicated to the left of the gel. (B) Table summarizing the identified homologues of the components of the mammalian UTX-MLL complex. Gene names, molecular weight in kDa, number of unique peptides and sequence coverage in percentage are reported. (C) Co-immunoprecipitations of WDR-5.1::HA and UTX-1. Total protein extracts from eggs of the indicated strains were immunoprecipitated using anti-HA affinity gel beads. The precipitates were analyzed by SDS-PAGE followed by western blotting using antibodies against HA, GFP or endogenous UTX-1. Input = 30 µg of protein extract. NBF = non bound fraction. (D) UTX-1-associated protein complex assessed by size exclusion chromatography. Superose 6 gel filtration of total protein extracts derived from UTX-1 mutant rescued with wild-type *(utx-1+UTX-1::GFP)* and catalytically inactive UTX-1 *(utx-1+UTX-1DD::GFP)*. Fractions were analyzed by western blotting using GFP and actin antibodies.

The mammalian UTX is part of the MLL3/MLL4 H3K4me3 methyltransferase complex [22]–[24] that also includes the specific component PTIP, and WDR5, ASH2L, and RbBP5 as core components, which are also shared by other complexes [25]. The high conservation of these proteins in nematodes (WDR5/*tag-125/wdr-5.1*, ASH2L/*ash-2*, RbBP5/*F21H12.1/rbbp-5*, MLL3-4/*set-16*, UTX/*utx-1*, and PTIP/*pis-1*), suggests that a similar complex could also exist in *C. elegans*. To test if an MLL3-4/UTX-like complex (SET-16/UTX-1) is present in *C. elegans*, we purified GFP-tagged UTX-1 and associated proteins from a mixed population of transgenic animals, enriched with embryos (Figure 5A). The identities of the interacting proteins were determined by mass spectrometry and are listed in the Table S1. As a control, N2 lysates were subject to the same procedure and the recovered proteins (listed in Table S2) were considered contaminants and used to confirm the specificity of the identified interacting proteins. Strikingly, all of homologous components of the mammalian MLL3/4 complex were identified as UTX-1 partners in *C. elegans* (Figure 5B). As further verification, we utilized a transgenic line carrying HA-tagged WDR-5.1 [26], the most prominent WDR5-like protein recovered by mass spectrometry, in which we expressed *UTX-1::GFP*. As shown in Figure 5C, in lysates derived from embryos, both *UTX-1::GFP* and endogenous UTX-1 were found associated with WDR5.1, further supporting the existence of a SET-16/UTX-1 complex in *C. elegans*. Importantly, the catalytically inactive mutant *UTX-1DD::GFP* was also recovered by WDR-5.1 immunoprecipitation (Figure 5C). Gel filtration analysis of lysates from transgenic lines carrying either the wild-type or the catalytically inactive forms of UTX-1 further confirmed that both UTX-1 and UTX-1DD are engaged in large complexes (Figure 5D), further supporting that a functional JmjC domain is not required for the association with the complex.

**Figure 6:**
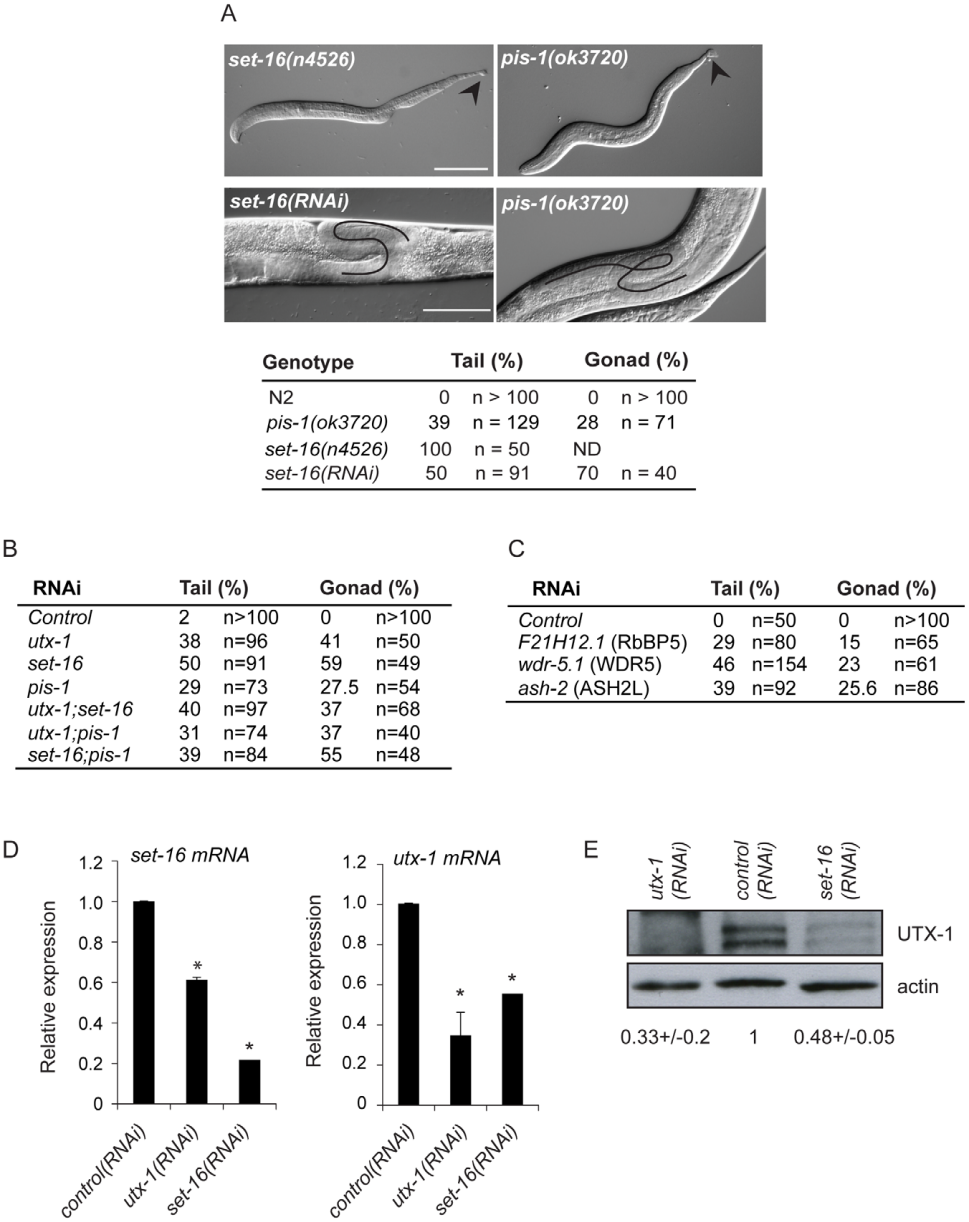
Functions of the SET-16/UTX-1 complex. (A) (Upper panel) DIC images of *set-16(n4526)* and *pis-1(ok3720)* mutant larvae. Arrowhead indicates misshapen tails. Scale bar is 50 µm. (Lower panel) DIC images of *set-16(RNAi)* and *pis-1(ok3720)* adults. Gonadal migration defects are shown. The black line indicates the aberrant gonadal migration. Scale bar is 20 µm. Animals are oriented anterior to the left. The percentage of tail and gonadal defects associated to loss or reduction of *set-16* and *pis-1* are reported on the right. (B) Percentage of tail and gonad defects after *RNAi* of the indicated genes. F1 L1 larvae and adult animals from at least two independent experiments were scored. (C) Percentage of tail and gonad defects after *RNAi* of the indicated genes. F1 or F2 L1 larvae and adult animals from at least two independent experiments were scored. (D) *utx-1* and *set-16* mRNA levels in embryos of worms treated with control, *utx-1* or *set-16(RNAi)*. *P<0.01 (Student's t-test). (E) Protein lysates of embryos from worms treated with control, *utx-1* or *set-16(RNAi*) were probed with an antibody against UTX-1. Actin was used as loading control. The signal was quantified using ImageJ program and normalized to actin. Indicated values are relative to *control (RNAi)* and derive from two independent experiments.

We then verified the functional correlation of the SET-16/UTX-1 complex components by testing if their loss or downregulation could result in phenotypes similar to those observed in the *utx-1* mutant. Loss of *set-16* results in embryonic and early larval lethality [27]. The analysis of *set-16(n4526)* young larvae revealed the presence of posterior defects similar to those identified in *utx-1* null animals (Figure 6A and Figure S8), and *set-16(RNAi)* animals that escaped embryonic and early larval lethality, often had abnormal gonad migration and enlargement (Figure 6A, 6B and Figure S9), which phenocopied the effect of the loss of *utx-1*. Similarly, in *pis-1(ok3720)* mutants and *pis-1(RNAi)* animals, posterior and gonadal defects were observed, although at a lower degree (Figure 6A and 6B, Figures S8 and S9). RNA interference of the core components of the complex (*F21H12.1*, *wdr-5.1*, and *ash-2*) also resulted in posterior and gonadal defects similar to the ones observed in *utx-1* mutants (Figure 6C and Figure S9). It should be noted, that enlargement of the proximal gonad was never observed after the reduction by RNAi of *F21H12.1* and *ash-2* and was rarely observed in *wdr-5 (RNAi)* animals (Figure S9). We then tested the effects of simultaneously downregulating specific components of the complex by *RNAi*. As shown in Figure 6B, the concurrent knockdown of *utx-1* and *set-16* or *pis-1* did not enhance the phenotypes; similar results were obtained with concomitant silencing of *pis-1* and *set-16*. The high degree of phenotypic similarity and the absence of redundancy are evidence that these genes are acting in the same genetic pathways to regulate posterior patterning and somatic gonadal development. Along the same line, qPCR analysis revealed that *set-16* downregulation by RNA interference results in a reduction of *jmjd-3.1* mRNA (about 60% decrease compared to control RNAi, data not shown), further supporting the notion that UTX-1 and SET-16 act in the same complex.

Since the catalytic activity of UTX-1 is not necessary to rescue the developmental defects observed both in *utx-1* mutants and in animals in which different factors of the complex were lost or down-regulated, we hypothesized that UTX-1 might regulate the expression of other components of the complex. In support of this, we found that the levels of *set-16* mRNA were reduced in *utx-1(RNAi)* animals (Figure 6D). Interestingly, downregulation of *set-16* also results in decreased expression of *utx-1* mRNA and protein (Figure 6D and 6E), suggesting an interdependent regulation of, at least, these two members of the complex.

Taken together the data demonstrate that the SET-16/UTX-1 complex is present in *C. elegans*, and it is required for development. That the loss or downregulation of single components of the complex results in similar phenotypes as those observed in *utx-1* null mutants, indicates that each component is required for the complex to function normally and that the defects associated with the loss of UTX-1 are likely the result of compromised SET-16/UTX-1 complex activity.

## Discussion

We have demonstrated that *C. elegans* UTX-1 is an H3K27me2/3 demethylase that is essential for development during embryonic and larval stages of the nematode, independently of its demethylase activity. Animals lacking the maternal and zygotic contribution of UTX-1 arrest during late embryogenesis. Although, analyses of reporter genes revealed no major defects in lineage specifications, a reduction of *myo-3::GFP*, expression, but not *hlh-1::GFP*, was observed in *utx-1* mutant animals, suggesting that *utx-1* might regulate genes involved in muscle function. In agreement, mammalian UTX has been implicated in terminal differentiation of muscle cells [28]. The maternal contribution of UTX-1 allows *utx-1(m+/z−)* worms to reach adulthood, but defects arise at different stages of development, including abnormal gonad migration and oocyte misorganization. This latter phenotype could explain, at least in part, the reduced fertility of *utx-1(m+/z−)* animals. We have previously shown that proper gonad migration partly depends on another H3K27me3 demethylase, *jmjd-3.1* [6]. The expression level of *jmjd-3.1* is significantly reduced in *utx-1(RNAi)* animals. However, it should be noted that the loss of *utx-1* leads to a more severe phenotype than the loss of *jmjd-3.1*, which only influences gonadal processes at high temperature and moderately reduces fertility. These results suggest that *utx-1*, in addition to *jmjd-3.1*, modulate additional genes required for establishing the correct developmental program of gonads.

While *utx-1* represents the unique UTX/UTY homologue, *C. elegans* has three other genes with homology to the single mammalian *JMJD3* gene (*jmjd-3.1*, *jmjd-3.2* and *jmjd-3.3*). We generated mutant animals carrying mutations in all three JMJD3-like genes and, unexpectedly, we did not detect any additional phenotypes in the triple mutants, other than the phenotypes already reported for *jmjd-3.1* [6]. While it is possible that residual gene function remains in these mutants, the global level of H3K27me3 was significantly increased in the triple knockout worms, whereas no increase was observed in the *jmjd-3.1* mutant strain alone ([6]; Figure 4B). This data suggests that the JMJD3-like demethylases might regulate the expression of restricted sets of genes or that they have overlapping functions. Our analysis of the global levels of H3K27me2/3 also suggests that UTX-1 is the most important demethylase for the removal of the H3K27me3 mark among the members of the KDM6 family. Accordingly, the loss of *utx-1* results in sterility (in *m+/z−* worms) and in embryonic lethality (in *m−/z−* worms) while animals lacking the three JMJD3 homologues are fertile and viable. This result indicates that *utx-1* plays unique and essential roles during embryonic and postembryonic development and suggests that the JMJD3-like proteins, like the human homologues [10], [29], are mainly required for regulating cellular responses under particular conditions, such as stress or aging.

Strikingly, we found that the catalytic activity of *C. elegans* UTX-1 is not required for the function of the protein in the developmental processes analyzed. This is at odds with a previous report describing the role of *utx1* genes in *D. rerio*, in which human wild-type, but not the catalytically inactive mutant, partially rescued the defects in UTX morphant animals [9]. We do not know if this apparent dissimilarity is due to an organismal difference, as suggested by the fact that *C. elegans* UTX-1 does not regulate HOX genes (data not shown) as it does in zebrafish [9] and that zebrafish has two UTX homologues, or to the different experimental approaches. Interestingly, recent results also suggest a catalytic-independent role for human JMJD3 and UTX in chromatin remodeling in a subset of T-box target genes [30]. Quantitative PCR and analysis of reporter genes failed, however, to identify any regulation of selected *C. elegans* T-box genes by UTX-1 (not shown).

The demonstration that UTX, which mediates H3K27me2/3 demethylase activity, is part of the MLL3/4 complex, which also has H3K4 methyltransferase activity [6], [7], suggests a model in which the coordinated removal of repressive marks (H3K27me3) and the deposition of activating marks (H3K4me3) fine-tune transcription during differentiation. We have shown that a similar complex is present in *C. elegans*, and that it is required to achieve proper development. Indeed, loss or reduction of each component of the complex results in phenotypes similar to those we observed in *utx-1* mutants. The lack of synergistic effects in double RNAi experiments further supports the notion that the components of the complex act in the same pathway(s) to regulate posterior body and somatic gonad development. Surprisingly, *utx-1* phenotypes are rescued by catalytically inactive UTX-1. The catalytically inactive mutant binds WDR-5.1 similarly to the wild-type protein, and it was identified in gel filtration experiments in a large complex, similarly to its wild-type counterpart. WDR-5.1 is also a component of other complexes and we cannot exclude at this time that the UTX-1/WDR-5.1 interaction might take place in the context of another complex. However, the components of other complexes with which WDR-5.1 is involved have, thus far, not been recovered by our mass spectrometry analysis. For example we did not identify the known WDR-5.1 binding partner SET-2 (the main H3K4me3 methyltransferase in *C. elegans*) [26], [31], [32], suggesting that UTX-1 is specifically recruited in the SET-16(MLL)-like complex.

Taken together these results strongly suggest that UTX-1 acts through a SET-16/UTX-1 complex and indicate that the primary role of UTX-1 in *C. elegans* development is independent of the demethylase activity, possibly through the regulation of expression of the complex components. This is suggested by our results showing that UTX-1 is, at least, required for the proper expression of *set-16*, and that SET-16 is required for the expression of *utx-1*, suggesting a positive feed forward mechanism for retaining the activity of the SET-16/UTX-1 complex. It is possible that there are additional functions for UTX-1; UTX-1 may be required for targeting the complex to specific genomic regions or it might play a role in the stability of the complex. To correctly address these possibilities, chromatin immunoprecipitation and mass spectrometry analysis must be performed in the context of *utx-1* null mutants. Unfortunately, these experiments are currently unfeasible since the *utx-1* mutant is unviable. It should be mentioned, however, that downregulation of the human UTX does not interfere with MLL complex formation (Agger K., Helin K., unpublished data), at least in mammals.

Finally, we do not know if UTX-1 works exclusively in association with the SET-16 complex or if it has additional roles as a single protein or in association with other complexes. The results obtained by mass spectrometry analysis suggest that this latter hypothesis might be correct. UTX-1 immunoprecipitates with other proteins involved in distinct chromatin complexes, such as HDA-1 and LIN-53, components of the NuRD complex [33], and MIX-1, which functions in the dosage compensation complex (DCC) [34]. While these interactions await further validation, it is worth noting that elements of the NuRD complex have been involved in vulva formation [33], a postembryonic event in which both UTX-1 and SET-16 have been implicated ([12] and data not shown), and that the DCC has been recently shown to interact with ASH-2, a member of the complex that we describe here. Therefore, it is conceivable that UTX-1 works in diverse chromatin complexes to accomplish functions required at different stages of development or under specific conditions. A MLL complex-independent role for UTX is also supported by the fact that a substantial amount of mammalian UTX is bound to promoter regions depleted of H3K4 methyl marks [35].

We did not detect reduced global levels of H3K4me3 in *utx-1* mutant animals (data not shown), which could be expected if UTX-1 regulates the function of a complex having H3K4 methyltransferase activity. This is in agreement with previously reported result in mammals and *C. elegans* [7], [12] and it is consistent with the fact that inactivation or downregulation of *set-16* only results in a minor, if any, reduction in global levels of H3K4me3 [12], [35], [36]. Indeed, similar to mammals, H3K4me3 deposition in *C. elegans* is mainly regulated by the other H3K4me3 methyltransferase, *set-2* [26], [36]. This observation suggests that the SET-16/UTX-1 complex regulates the mark deposition only for a subset of genes, and, consequently, complex impairment does not impact the global levels H3K4me3.

Our analysis failed to uncover a role for UTX-1 catalytic activity during development, and a major question is therefore whether this activity is required for any biological function in *C. elegans*. Since this work focused on the role of UTX-1 during development, the catalytic activity might be required for other processes that are not implicated in developmental programs and are dispensable for viability. Indeed, recent reports implicate the catalytic activity of UTX-1 in aging [13], [18]. Moreover, UTX-1 activity could act during germline formation to counteract the well-established role of the PRC2/MES complex [37]–[43]. However, we have thus far not been able to establish a function of UTX-1 during germline formation neither alone nor in synthetic interaction with components of the MES complex (data not shown).

In summary, we have shown that UTX-1 plays an essential role in several developmental processes in *C. elegans*. Surprisingly, the catalytic activity is dispensable for proper development, and our data suggest that UTX-1 acts, instead, through a SET-16/UTX-1 complex. Future studies will be directed at identifying the specific target genes regulated by the complex and the possible role that UTX-1 might play in the stability of the complex and in its recruitment to the target genes.

## Materials and Methods

### Genetics and strains

*C. elegans* strains were cultured using standard methods [44]. Strains used were as follows: wild-type Bristol (N2), *utx-1(tm3118)X*, *utx-1(tm3136)X*, *jmjd-3.1(gk384)X*, *jmjd-3.2(tm3121)X*, *jmjd-3.3(tm3197)X*, JK2049 *qls19 V*, *set-16(n4526)III*, *pis-1(ok3720)IV, AZ217(myo-2::GFP), MS438(elt-2::GFP), GS3798(dpy-7::YFP), OP64(hlh-1::GFP), PS3729(ajm-1::GFP)*. The strain *wdr-5.1/tag-125::HA* and *OE4201(myo-3::GFP)* were generous gifts from Francesca Palladino and Thomas Bürglin, respectively. Transgenic animals with specific genetic backgrounds were generated by standard crossing procedure. The *C. elegans utx-1* sequence is located on chromosome X and the transcript encompasses 14 exons coding for a predicted protein of 1168 amino acids. The ATG of the gene is located at position 13888 bp of the D2021 cosmid (U23513) and a TAG terminator codon at position 19549 bp. Two alleles of *utx-1* were identified at the National BioResource Project (NBRP), Japan. Both alleles were backcrossed three times with N2 before the phenotypic analysis and maintained in culture as heterozygotes. The *tm3136* allele lacks 236 bp and the deletion is found at position 13920–14155 bp of the Genbank entry U23513. This deletion creates a premature stop codon and the deleted gene could potentially encode for a truncated protein of 28 amino acids. The *tm3118* is an out–of-frame deletion of 547 bp situated at position 17361–17907 bp of the Genbank entry U23513. The truncated protein potentially retains the first 620 amino acids and lacks the JmjC domain. Phenotypic analyses of *utx-1* mutant animals (*tm3136* and *tm3118*) were done in blind, before genotyping. *jmjd-3.2(tm3121)X* and *jmjd-3.3(tm3197)X* were backcrossed three times before analysis and their deletions, described in Wormbase, were confirmed by sequencing. KDM6 members are all positioned on the X-chromosome, with *utx-1* located very closely to *jmjd-3.1*, thus precluding the generation of a quadruple mutant lacking all the four H3K27me3 demethylases. The triple mutant with deletions in *jmjd-3.1(gk384)X, jmjd-3.3(tm3197)X* and *jmjd-3.2(tm3121)X* was generated using standard crossing methods. The triple mutant *jmjd-3.2(tm3121);jmjd-3.3(tm3197);utx-1(tm3118)+UTX-1DD::GFP* (expressing UTX-1DD as an extrachromosomal array) was generated using standard crossing methods.

### Brood size

Single fourth-stage (L4) larvae were plated in agar plates with OP50 bacteria and moved to a new plate every 24 h. Viable progeny were counted every day, for 4 days at 25°C. The average number of progeny produced by a single animal is reported.

### RNA interference (RNAi)

*RNAi* was performed by feeding and carried out as described previously [45]. For UTX-1, a clone (X-4I10) containing the region from 18167 bp to 19214 bp of the GenBank entry U23513 (c in Figure 1A), was obtained from the *C. elegans RNAi* feeding library (J. Ahringer's laboratory, Wellcome Trust/Cancer Research UK Gurdon Institute, University of Cambridge, Cambridge, UK). Two other clones (a and b in Figure 1A), spanning the regions 14109–14331 bp and 17512–18014 bp of the GenBank entry U23513, respectively, were constructed by PCR. We generated RNAi clones for *set-16*, *ash-2*, *pis-1*, *tag-125*, and *F21H12.1* by amplifying cDNA fragments (approximately 500 pb) before cloning in L4440 plasmid using *EcoRI* restriction sites (all primer sequences available upon request). Eggs, prepared by hypochlorite treatment, were added onto *RNAi* bacteria-seeded NMG plates and cultivated at 25°C. Control animals were fed with bacteria carrying the control vector (L4440). Generally, F1 progeny was scored for phenotypes.

### Real-time quantitative PCR (RT–qPCR)

Total RNA was isolated from eggs using TRIzol reagent (Invitrogen) and RNAeasy Minikit (Qiagen). cDNA was synthesized using reagents from the TaqMan Reverse Transcription kit (Applied Biosystems). qPCR was performed using SYBR Green 2× PCR Master mix (Applied Biosystems) in an ABI Prism 7300 Real Time PCR system (Applied Biosystems). The measures were normalized to ribosomal protein (*rpl-26*) RNA levels. All reactions were performed in triplicate, in at least three independent experiments. All primer sequences are available upon request.

### Quantitative Western blot analysis

For mutant strains, total protein extracts were prepared from eggs obtained by hypochlorite treatment of adults grown on OP50 at 25°C. For RNAi-treated animals, extracts were prepared from eggs obtained by hypochlorite treatment of adults grown on HT115 containing either the empty feeding vector, or specific *RNAi*. Protein concentration was estimated using the modified micro-Lowry assay and equal amounts of protein were loaded. The following antibodies were used: polyclonal anti-H3 (Abcam 1791, lot GR9204-1) 1∶30000; polyclonal anti-H3K27me1 (Upstate 07-448, lot DAM1598790) 1∶5000, polyclonal anti-H3K27me2 (Abcam 24684, lot 956943) 1∶2000; polyclonal anti-H3K27me3 (Upstate 07-449, lot 701050758) 1∶2000; monoclonal anti-actin (Chemicon International MAB1501) 1∶10000; peroxidase-labeled anti-rabbit and anti-mouse secondary antibodies (Vector). The specificity of H3K27 antibodies has been tested as shown in Figure S12. Polyclonal *C. elegans* UTX-1 antibodies were obtained through the Eurogentec polyclonal antibody production service. To generate a specific UTX-1 antiserum, rabbits were immunized with two UTX-1 peptides (MDESEPLPEERHPGNC and SYRRSYKDDANRLDHC). Antibodies were purified using affinity columns coupled with the same peptides and used at 1∶500 dilution. The antibody recognizes in the lysate of wild-type animals a specific band of the predicted size of 134 kDa, absent in the lysates obtained from *utx-1* mutant alleles (Figure S12D). Western blots were quantified using ImageJ program (National Institutes of Health).

### Construction of tagged *UTX-1* and *UTX-1DD*

For the *UTX-1::GFP* construct, a 6956-bp fragment of *utx-1* including 1290 bp of promoter region and the entire coding region was PCR-amplified from N2 genomic DNA. The resulting fragment was inserted in the multiple cloning site of the pPD95.75 vector (Fire lab).

For the *UTX-1DD::GFP* construct, the *UTX-1::GFP* construct was mutated using the QuikChange Site-Directed Mutagenesis Kit (Stratagene). Specifically, the DNA sequence was mutated so that the histidine at position 914 (H914) and the aspartic acid at position 916 (D916) were changed to alanine. The DNA sequences of both constructs were verified by sequencing.

### Microinjection and production of transgenic lines

To obtain lines carrying extra-chromosomal arrays, 20 ng/µl of *UTX-1::GFP* and *UTX-1 DD::GFP* constructs were each co-injected with 100 ng/µl pRF4(rol-6(su1006)) or ttx-3p::RFP in wild-type N2 worms (*N2+UTX-1::GFP* and *N2+UTX-1DD::GFP*). Transgenic lines in *utx-1(tm3136 and tm3118)* genetic backgrounds (*utx-1+UTX-1::GFP and utx-1+UTX-1DD::GFP*) were generated by crossing.

### Microscopy, image processing, quantification, and statistical analysis

Fluorescence microscope and DIC pictures were acquired using an Axiovert 135, Carl Zeiss, Inc. with a 63× Plan Apochrome objective with a NA of 1.4 in immersion oil and a 40× Plan NEOFLUAR with a NA of 0.75, respectively. Pictures were taken at room temperature with a CoolSNAP cf2; Photometrics camera. All pictures were exported in preparation for printing using Photoshop (Adobe). MetaMorph software (MDS Analytical Technologies) was used to quantify the mean and s.e.m. of integrated intensities per cell as described in [46]. The 2–4 most anterior intestinal cells were used for the quantification of H3K27me3/GFP and more than 20 cells from at minimum of 10 animals for each genotype (*N2+UTX-1::GFP* , *N2+UTX-1DD::GFP* and GFP-negative siblings) were analyzed in two independent experiments. Only animals showing good H3K27me3 signal in the gonads, as indication of successful immunostaining, were used for quantification. Statistical calculations were performed using the Graphpad Prism software package (GraphPad Prism version 4.00 for Windows, GraphPad Software, San Diego California USA, www.graphpad.com). Distribution of data was assessed using three different normality tests: KS normality test, D'agostino & Pearson Normality test and Shapiro-Wilk normality test. When data were normally distributed according to these tests, parametric statistics were applied (t-test), otherwise non-parametrical statistical analysis (Mann-Whitney U test) was performed. When comparing more than two groups ANOVA tests were applied. For all tests p≤0.05 was considered significant.

### Immunofluorescence

For immunostaining, animals were fixed and permeabilized as described [47]. Polyclonal anti-H3K27me3 (Upstate 07-449) and polyclonal anti-UTX-1 (Eurogentec, clone 3917, this study) were used. Secondary antibodies were: goat anti-mouse IgG (Alexafluor 488); goat anti-rabbit IgG (Alexafluor 594), both purchased from Invitrogen. DAPI (Sigma, 2 ug/ul) was used to counter-stain DNA. Eggs immunofluorescence was performed by freeze crack method, adding eggs to polylysine treated slides. After freezing at −80°C for 30 minutes, the cover slip was removed and embryos were fixed in methanol at −20°C for 10 min. Primary antibody was incubated overnight at 4°C in a humid chamber and secondary antibody was incubated 1 h at room temperature. Washes were in PBS/tween 0.2%. Mounting medium for fluorescence with DAPI (Vectashield H1200) was used.

### GFP pulldown

Generation of transgenic strain *utx-1+UTX-1::GFP* has been described earlier in Materials and Methods. Total protein extracts was obtained by grinding a frozen pellet of mixed eggs and adults with a mortar and pestle into powder, the latter was resuspended in IP buffer containing 300 mM KCl, 0.1% Igepal, 1 mM EDTA, 1 mM MgCl_2_, 10% glycerol, 50 mM Tris HCl (pH 7.4) and protease inhibitors. GFP-Trap beads (Chromotek) were used to precipitate GFP-tagged proteins from this lysate. Approximately 200 mg of total proteins was used for the pulldown in IP buffer. Following incubation and washes with the same buffer, proteins were eluted with acidic glycine (0.1 M [pH 2.5]), resolved on a 4–12% NuPage Novex gel (Invitrogen), and stained with Imperial Protein Stain (Thermo Scientific). The gel was sliced into 21 bands across the entire separation range of the lane. Cut bands were reduced, alkylated with iodoacetamide, and in-gel digested with trypsin (Promega) as described previously [48], prior to LC/MS-MS analysis.

### Mass spectrometry of proteins

Peptide identification was performed on an LTQ-Orbitrap mass spectrometer (Thermo Fisher Scientific, Germany) coupled with an EASY-nLC nanoHPLC (Proxeon, Odense, Denmark). Samples were loaded onto a 100 µm ID×17 cm Reprosil-Pur C18-AQ nano-column (3 µm; Dr. Maisch GmbH, Germany). The HPLC gradient was from 0 to 34% solvent B (A = 0.1% formic acid; B = 95% MeCN, 0.1% formic acid) over 30 minutes and from 34% to 100% solvent B in 7 minutes at a flow-rate of 250 nL/min. Full-scan MS spectra were acquired with a resolution of 60,000 in the Orbitrap analyzer. For every full scan, the seven most intense ions were isolated for fragmentation in the LTQ using CID. Raw data were viewed using the Xcalibur v2.1 software (Thermo Scientific). Data processing was performed using Proteome Discoverer beta version 1.3.0.265 (Thermo Scientific). For database search we included both Mascot v2.3 (Matrix Science) and SEQUEST (Thermo Scientific) search engines. Database of *C. elegans* protein sequences was downloaded from Uniprot. Trypsin was selected as digestion enzyme and two missed cleavages were allowed, carbamidomethylation of cysteines was set as fixed modification and oxidation of methionine as variable modification. MS mass tolerance was set to 10 ppm, while MS/MS tolerance was set to 0.6 Da. Peptide validation was performed using Percolator and peptide false discovery rate (FDR) was set to 0.01. For additional filtering, maximum peptide rank was set to 1 and minimum number of peptides per protein was set to 2. Protein grouping was performed, in order to avoid presence of different proteins identified by non-unique peptides. We manually investigated whether the protein listed to represent the protein group was the most characterized in terms of sequence coverage and number of peptides identified.

### Protein interaction assay

For co-immunoprecipitation assays, frozen eggs (prepared by hypochlorite treatment) were reduced into powder using a mortar and pestle. The powder was resuspended in IP buffer (described in GFP pulldown section) and 5–10 mg was incubated with Protein G agarose beads (Upstate) overnight at 4°C. Soluble fraction was collected and incubated with EZview anti-HA affinity gel beads (Sigma Aldrich) during 2 h at 4°C. The immunoprecipitates and the protein G beads were washed five times in IP buffer, boiled in SDS-sample buffer and analyzed by SDS-PAGE followed by western blotting. Antibodies used in those experiments were: anti-HA (Covance HA.11, clone 16B12), anti-GFP (Roche, 11814460001) and anti-UTX-1 (Eurogentec, clone 3917, this study). Quantification of western blots was performed using ImageJ program (National Institutes of Health, USA).

### Analytical gel filtration chromatography

Eggs from indicated strains were grinded to powder, resupended in IP buffer (50 mM Tris-HCl pH 7.4, 300 mM KCl, 1 mM MgCl_2_, 1 mM EDTA, 0.1% Igepal and complete protease inhibitors [Roche]) and incubated on wheel for 30 min at 4°C. Protein extracts were recovered by centrifugation at 20,000 *g*, 30 min at 4°C and clarified by ultracentrifugation at 627,000 *g* for 30 min at 4°C. Fresh extracts were fractionated on a Superose 6 HR 10/300 GL column (GE Healthcare) equilibrated in IP buffer. Size exclusion chromatography was performed on a fast protein liquid chromatography (FPLC) system and an ÄKTA purifier (GE Healthcare). Elution profiles of blue dextran (2,000 kDa), thyroglobulin (660 kDa) and bovine serum albumin (66 kDa) were used for calibration. Fractions of 1 ml were collected and precipitated with 25% trichloroacetic acid and then centrifuged at 20,000 *g* for 10 min at 4°C. Pellets were washed two times in cold acetone, air dried, and resuspended in loading buffer for Western blot analysis.

## Supporting Information

Figure S1 Embryonic/larval defects in *utx-1* mutant and *ajm-1::GFP* analysis. (A) DIC (left) and epifluorescence (right) images of *utx-1(tm3136)* rescued (right animal) or not (left animal) with a translational reporter of *utx-1*, under control of its own promoter. Note the morphological defects in the not-rescued animal. (B) *ajm-1::GFP* localization in *utx-1(tm3136)* embryos rescued (right, note the nuclear staining of UTX-1::GFP) or not (left) with a translational reporter of *utx-1*, under control of its own promoter. *ajm-1::GFP* is correctly localized at initial stages but appear disorganized at later stages (3 fold and L1) in not-rescued *utx-1* mutant. Bars are in A 50 µm, in B 20 µm.
(TIF)

Figure S2 *elt-2::GFP* analysis in *utx-1* mutant. Pattern of expression of *elt-2::GFP* in N2 and in *utx-1(tm3136)* allele at different embryonic stages and L1. Bars are 20 µm.
(TIF)

Figure S3 *myo-2::GFP* analysis in *utx-1* mutant. Pattern of expression of *myo-2::GFP* in N2 and in *utx-1(tm3136)* allele at different embryonic stages and L1. Bars are 20 µm.
(TIF)

Figure S4 *hlh-1::GFP* analysis in *utx-1* mutant. Pattern of expression of *hlh-1::GFP* in N2 and in *utx-1(tm3136)* allele at different embryonic stages and L1. Bars are 20 µm.
(TIF)

Figure S5 *dpy-7::GFP* analysis in *utx-1* mutant. Pattern of expression of *dpy-7::GFP* in N2 and in *utx-1(tm3136)* allele in L1. The staining in the pharynx is due to a co-injection marker. Bars is 20 µm.
(TIF)

Figure S6 *myo-3::GFP* analysis in *utx-1* mutant. Pattern of expression of *myo-3::GFP* in N2 and in *utx-1(tm3136)* allele at different embryonic stages and L1. Note the decreased level of *myo-3::GFP* in *utx-1* mutant compared to N2. Bars are 20 µm.
(TIF)

Figure S7 Efficiency of RNAi and brood size in *utx-1(RNAi)* treated worms. (A) Relative expression of *utx-1* mRNA after feeding *RNAi* treatments using three constructs targeting different regions of *utx-1*, indicated in Figure 1A. Level of UTX-1 protein after *utx-1(RNAi)* was measured by Western blot, using a specific antibody against UTX-1. Actin was used as loading control. The signals were quantified using ImageJ program and normalized to actin. Values are relative to *control RNAi*. Asterisks indicate results different at p<0.01 (Student's t-test). (B) Average number of eggs laid by worms treated with control (white bars) or *utx-1(RNAi)* (black bars).
(TIF)

Figure S8 Tail defects associated to loss/downregulation of specific components of the SET-16/UTX-1 complex. Top panels. Representative DIC images of posterior defects observed upon downregulation of *utx-1, set-16 and pis-1* using *RNAi*. F1 L1 are shown. Bottom panels. Representative DIC images of posterior defects observed in *utx-1(tm3136), set-16(n4226) and pis-1(ok3720)*. L1 larvae are shown. Bars are 10 µm.
(TIF)

Figure S9 Somatic gonad defects associated to loss/downregulation of the components of SET-16/UTX-1 complex. Representative DIC images of gonadal defects (top: aberrant migration, bottom: oocyte accumulation) observed in *utx-1(tm3136), pis-1(ok3720)* and after RNAi of the indicated genes. *set-16(n4226)* dies before gonad migration. Asterisks indicate oocytes accumulation at the distal region of the gonad, black lines indicate the aberrant gonadal migration. Accumulation of oocytes is not observed after down-regulation of *ash-2* and *wdr-5*. In RNAi, F1 or F2 young adult were scored.
(TIF)

Figure S10 The overexpression level of UTX-1 inversely correlates with H3K27me3 intensity. Level of H3K27me3 in N2 overexpressing wild-type *UTX-1::GFP*. H3K27me3 and GFP intensities in single intestinal cells (shown in Figure 3B) were analyzed as described in Materials and Methods. Scatter plot presentation of H3K27me3 levels in cells having low (n = 10, range: 0–14807 arbitrary units) medium (n = 11, range: 28645–145977 arbitrary units) or high (n = 11, range: 148852–258798 arbitrary units) levels of *UTX-1::GFP*. Statistical significance levels are provided. Worms analyzed were from two independent experiments. Mean of H3K27me3 is expressed in arbitrary units.
(TIF)

Figure S11 Expression of the KDM6 family members. (A) Relative expression of the KDM6 class members in wild-type animals at different stages, normalized to *rpl-26* levels. (B) Quantification of the western blot shown in Figure 4B. Bands were analyzed using ImageJ program and the values reported are relative to N2 levels. Note the increase level of H3K27me3 in the triple mutant. (C) Expression of transcriptional fusion constructs of the four members of the KDM6 family obtained as described in Text S1. Epifluorescence of adult animals is shown. Animals are oriented head to the left, ventral down. Bar is 100 µm. (D) Expression of the JMJD3-like genes in the indicated genetic backgrounds. The levels are relative to N2 and normalized to *rpl-26*.
(TIF)

Figure S12 Specificity of the antibodies. A) List of the antibodies used in this study. Specificity, reference, lot number and working dilution of the antibodies are indicated. (B) Specifity of the antibodies used in this study. Dot blots were performed as indicated in Text S1 and Methods and using the following peptides (indicated on the top): Histone H3 di-methyl K9 (H3K9me2), Histone H3 tri-methyl K9 (H3K9me3), Histone H3 mono-methyl K27 (H3K27me1), Histone H3 di-methyl K27 (H3K27me2), Histone H3 tri-methyl K27 (H3K27me3). Different amounts of peptides, ranging from 100 to 1 ng, as indicated on the right side of the blots, were spotted on the membrane before probing with the indicated antibodies (on the left). Two exposures of the same membrane are shown. (C) Efficiency of the antibodies used in this study on worm samples. Different amounts of worm total protein extracts (from 1 to 40 µg) were resolved on a 15% polyacrylamide gel and probed with the indicated antibodies. The western blots indicate that our analysis was performed using the linear range of the antibodies. (D) Specificity of the UTX-1 antibody. Total protein extracts (50 µg) from N2 and *utx-1* mutants were analyzed by Western blot using UTX-1 polyclonal antibody. Ponceau's staining of the membrane shows equal loading. Arrowhead indicates the UTX-1 band.
(TIF)

Table S1 List of proteins identified by GFP-IP/MS from UTX-1:GFP worm sample. Accession numbers and short descriptions of the identified proteins are given together with the number of peptides (Σ# peptides) recovered for each protein and the protein coverage (Σ coverage). M to AG columns indicate the 21 slices of the gel (with A = top of the gel and U = bottom of the gel).
(XLSX)

Table S2 List of proteins identified by GFP-IP/MS from N2 worm sample. Accession numbers and short descriptions of the identified proteins are given together with the number of peptides (Σ# peptides) recovered for each protein and the protein coverage (Σ coverage). M to AG columns indicate the 21 slices of the gel (with A = top of the gel and U = bottom of the gel).
(XLSX)

Text S1 Supporting Materials and Methods.
(DOCX)
