# Supplementary figures and images for: The C. elegans H3K27 Demethylase UTX-1 Is Essential for Normal Development, Independent of Its Enzymatic Activity
Source: PLoS Genet. 2012 May 3;8(5):e1002647. doi: 10.1371/journal.pgen.1002647 (PMC3342935; doi:10.1371/journal.pgen.1002647)

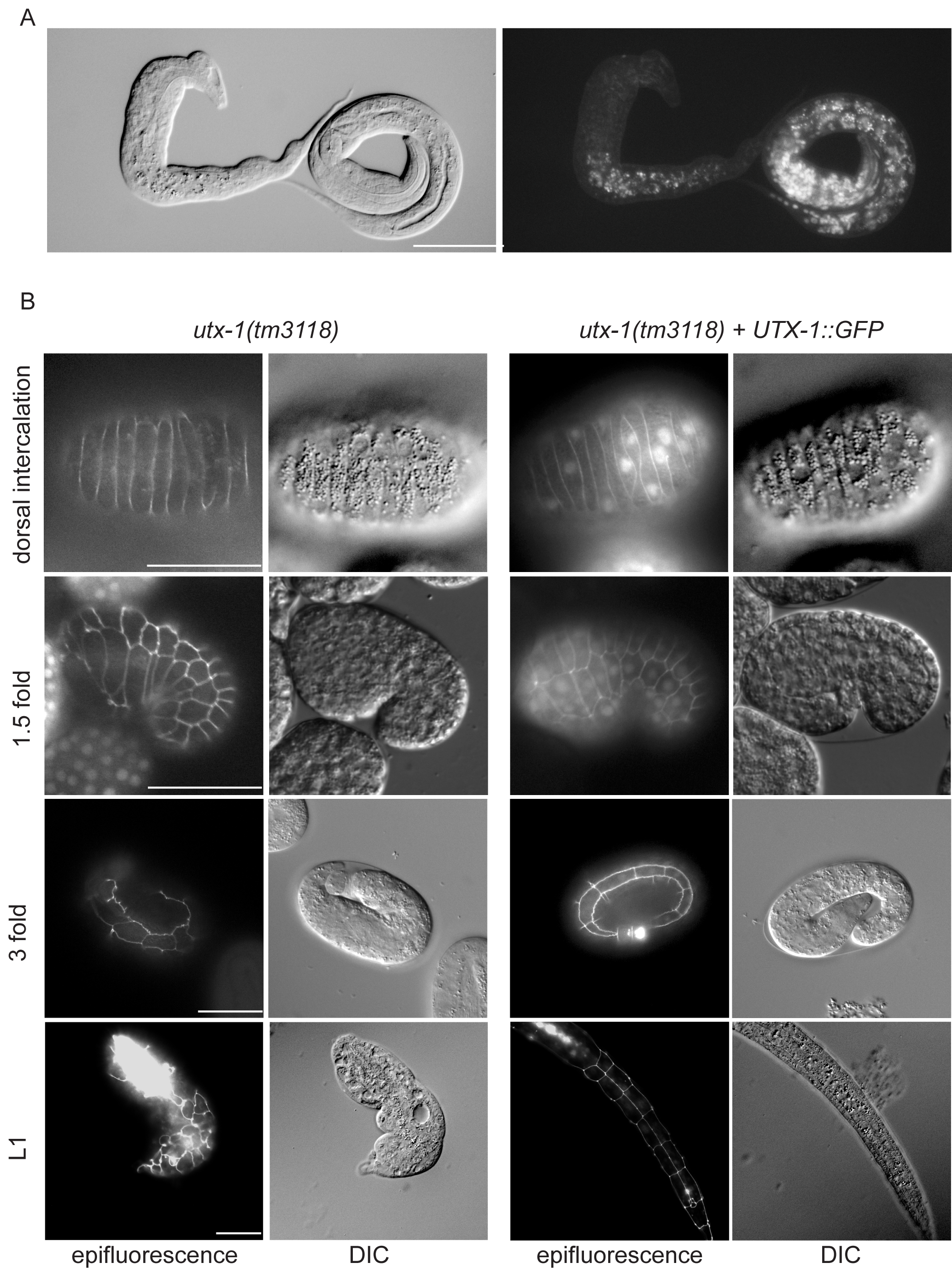

Supplement: Figure S1 — Embryonic/larval defects in utx-1 mutant and ajm-1::GFP analysis. (A) DIC (left) and epifluorescence (right) images of utx-1(tm3136) rescued (right animal) or not (left animal) with a translational reporter of utx-1, under control of its own promoter. Note the morphological defects in the not-rescued animal. (B) ajm-1::GFP localization in utx-1(tm3136) embryos rescued (right, note the nuclear staining of UTX-1::GFP) or not (left) with a translational reporter of utx-1, under control of its own promoter. ajm-1::GFP is correctly localized at initial stages but appear disorganized at later stages (3 fold and L1) in not-rescued utx-1 mutant. Bars are in A 50 µm, in B 20 µm. (TIF) [file pgen.1002647.s001.tif]

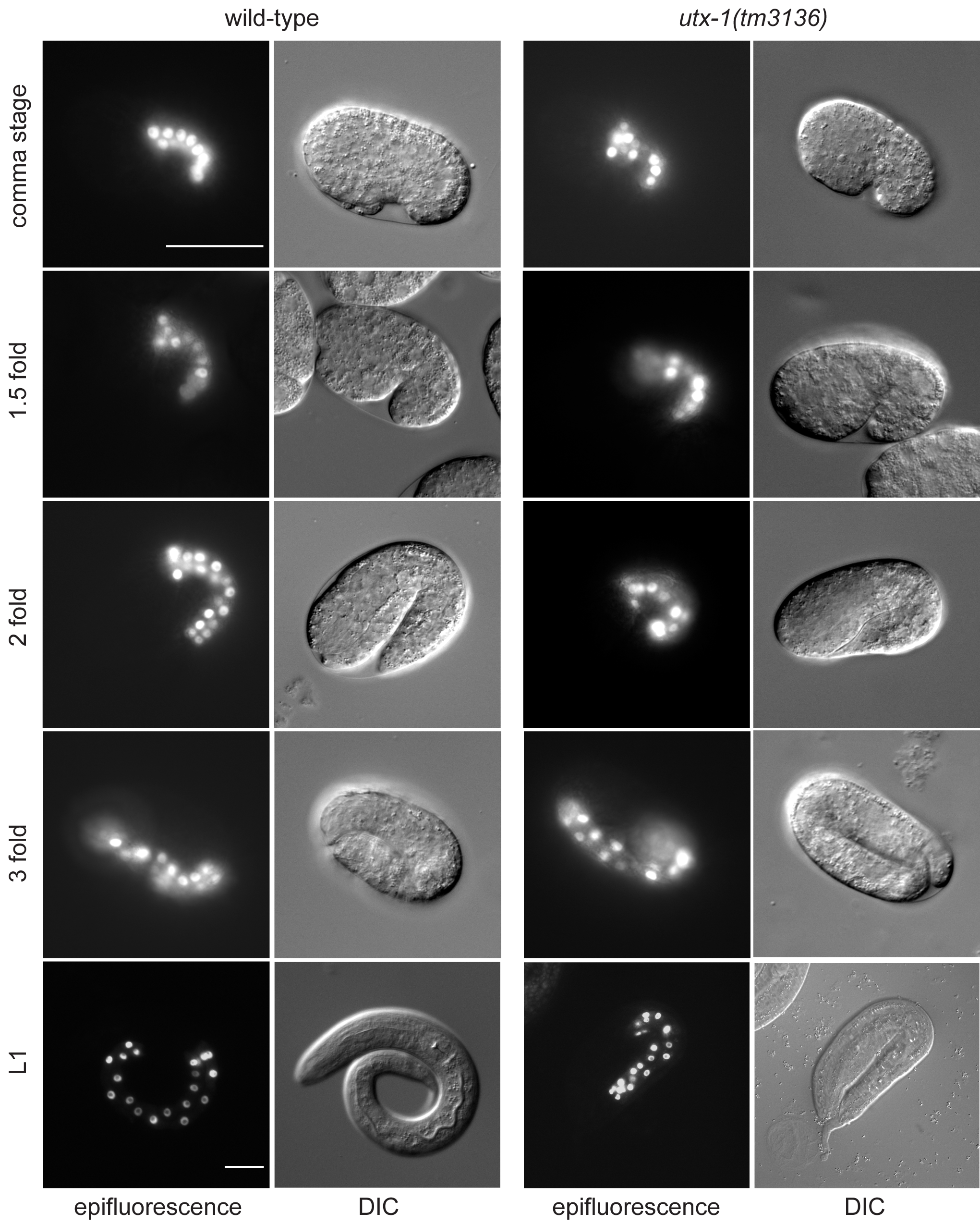

Supplement: Figure S2 — elt-2::GFP analysis in utx-1 mutant. Pattern of expression of elt-2::GFP in N2 and in utx-1(tm3136) allele at different embryonic stages and L1. Bars are 20 µm. (TIF) [file pgen.1002647.s002.tif]

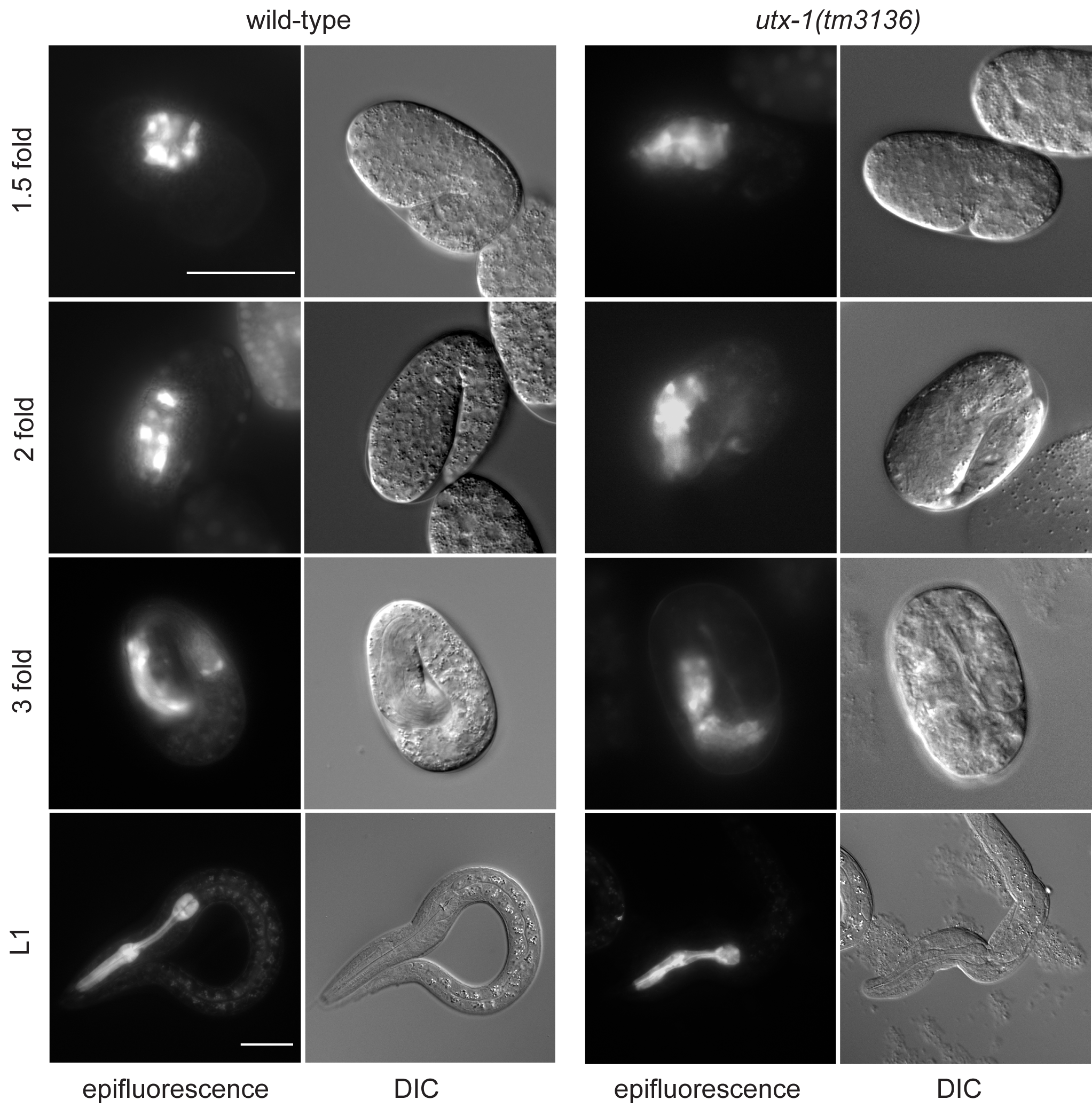

Supplement: Figure S3 — myo-2::GFP analysis in utx-1 mutant. Pattern of expression of myo-2::GFP in N2 and in utx-1(tm3136) allele at different embryonic stages and L1. Bars are 20 µm. (TIF) [file pgen.1002647.s003.tif]

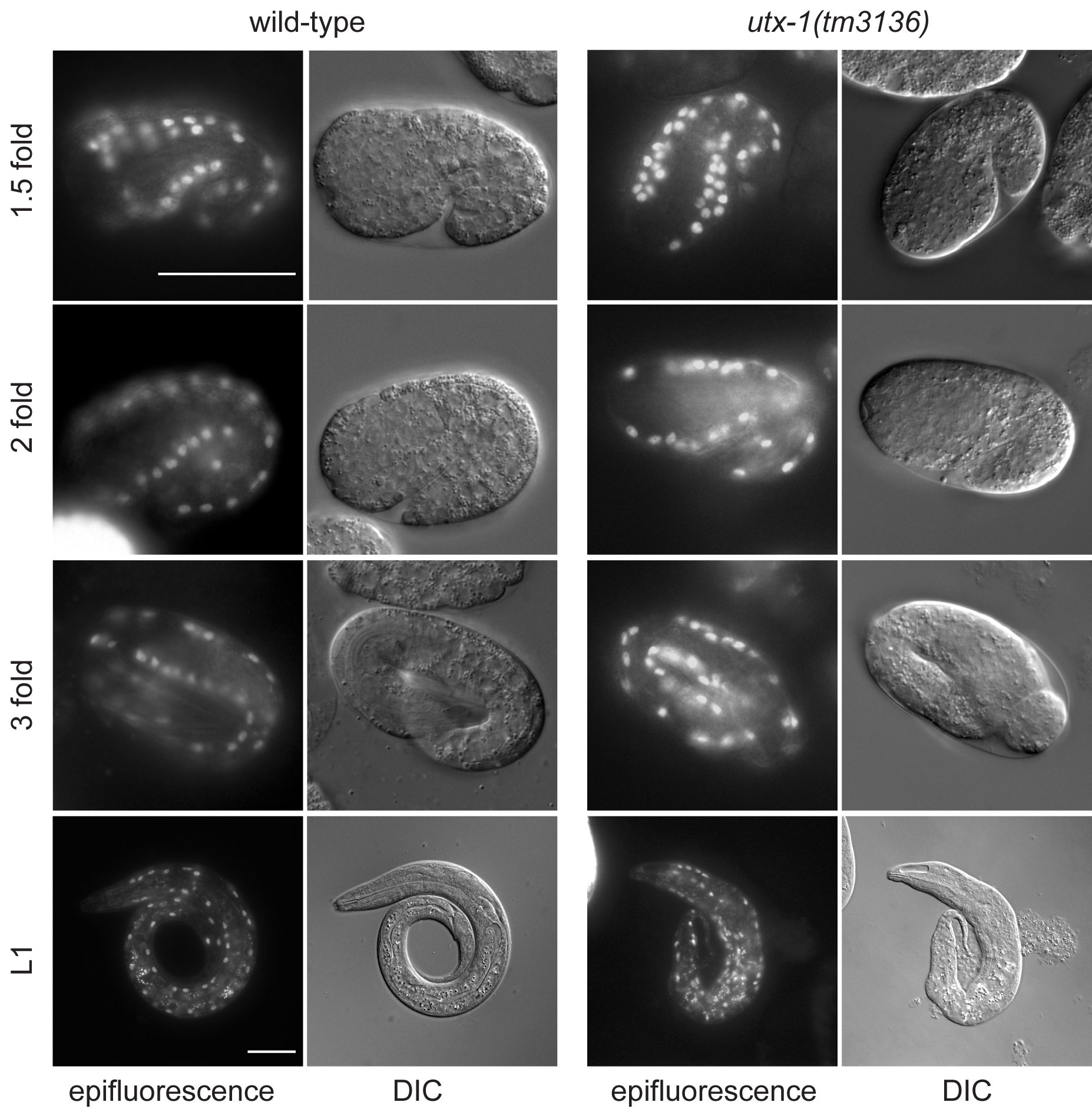

Supplement: Figure S4 — hlh-1::GFP analysis in utx-1 mutant. Pattern of expression of hlh-1::GFP in N2 and in utx-1(tm3136) allele at different embryonic stages and L1. Bars are 20 µm. (TIF) [file pgen.1002647.s004.tif]

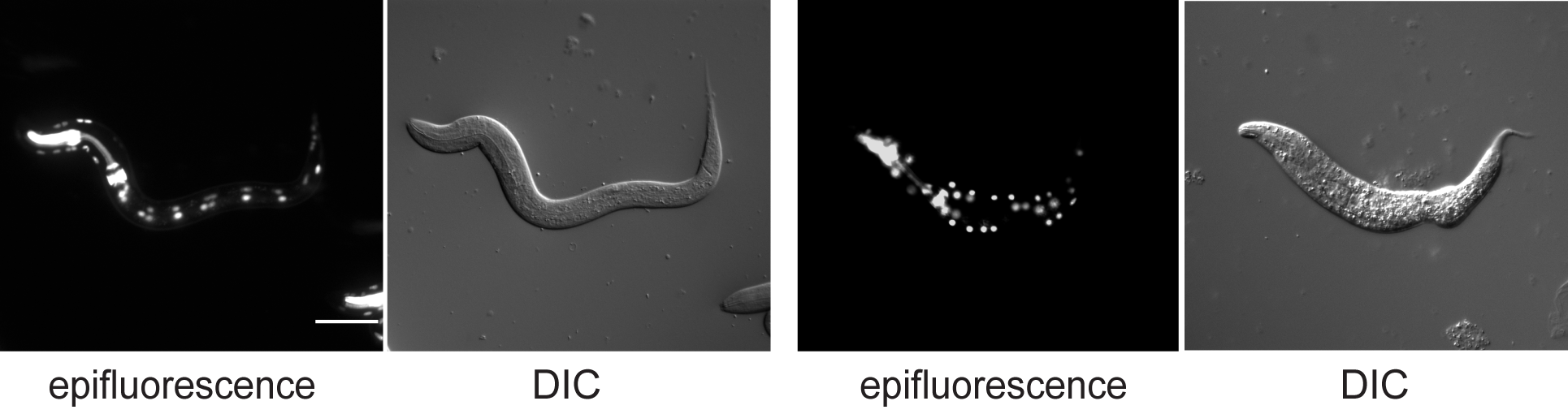

Supplement: Figure S5 — dpy-7::GFP analysis in utx-1 mutant. Pattern of expression of dpy-7::GFP in N2 and in utx-1(tm3136) allele in L1. The staining in the pharynx is due to a co-injection marker. Bars is 20 µm. (TIF) [file pgen.1002647.s005.tif]

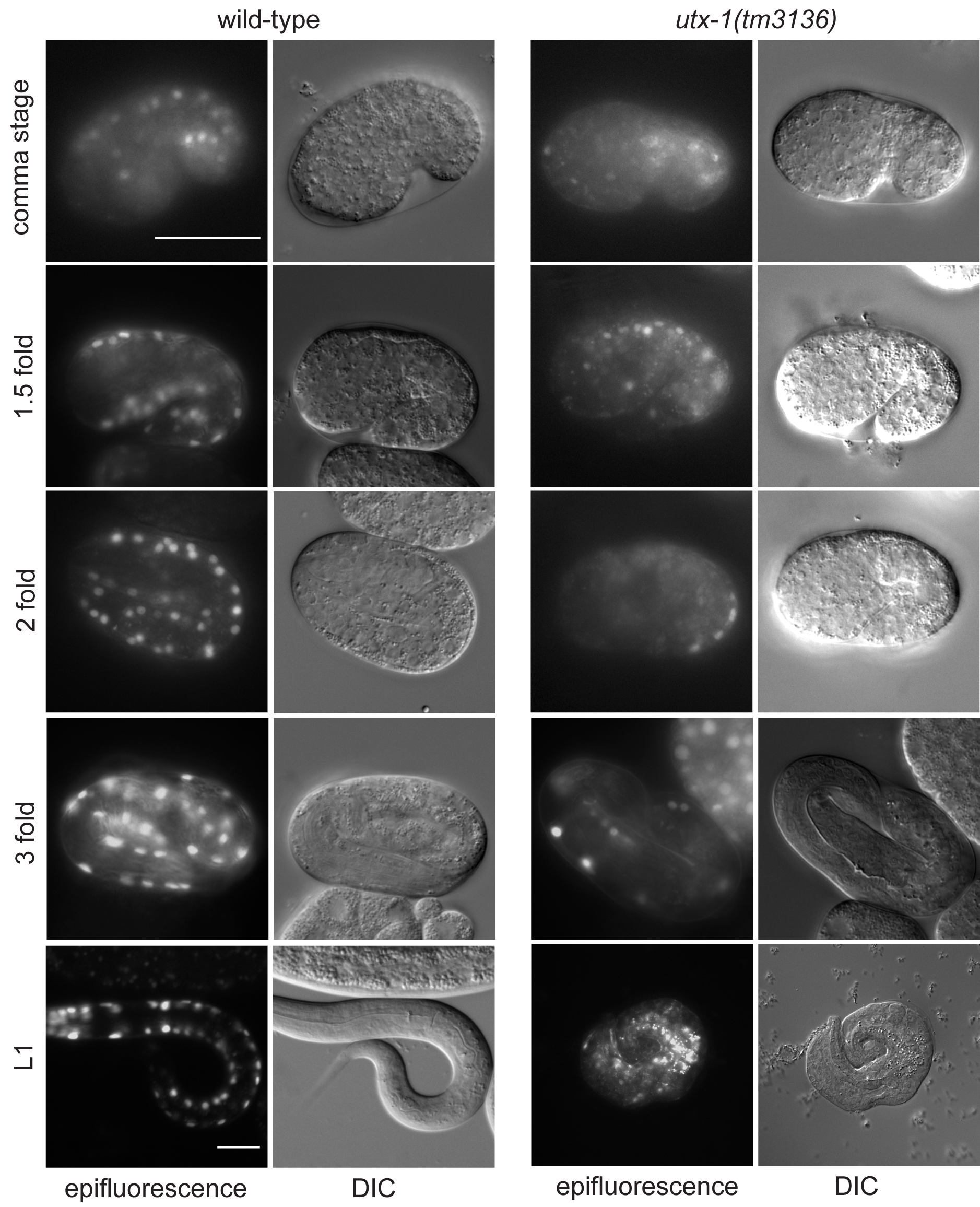

Supplement: Figure S6 — myo-3::GFP analysis in utx-1 mutant. Pattern of expression of myo-3::GFP in N2 and in utx-1(tm3136) allele at different embryonic stages and L1. Note the decreased level of myo-3::GFP in utx-1 mutant compared to N2. Bars are 20 µm. (TIF) [file pgen.1002647.s006.tif]

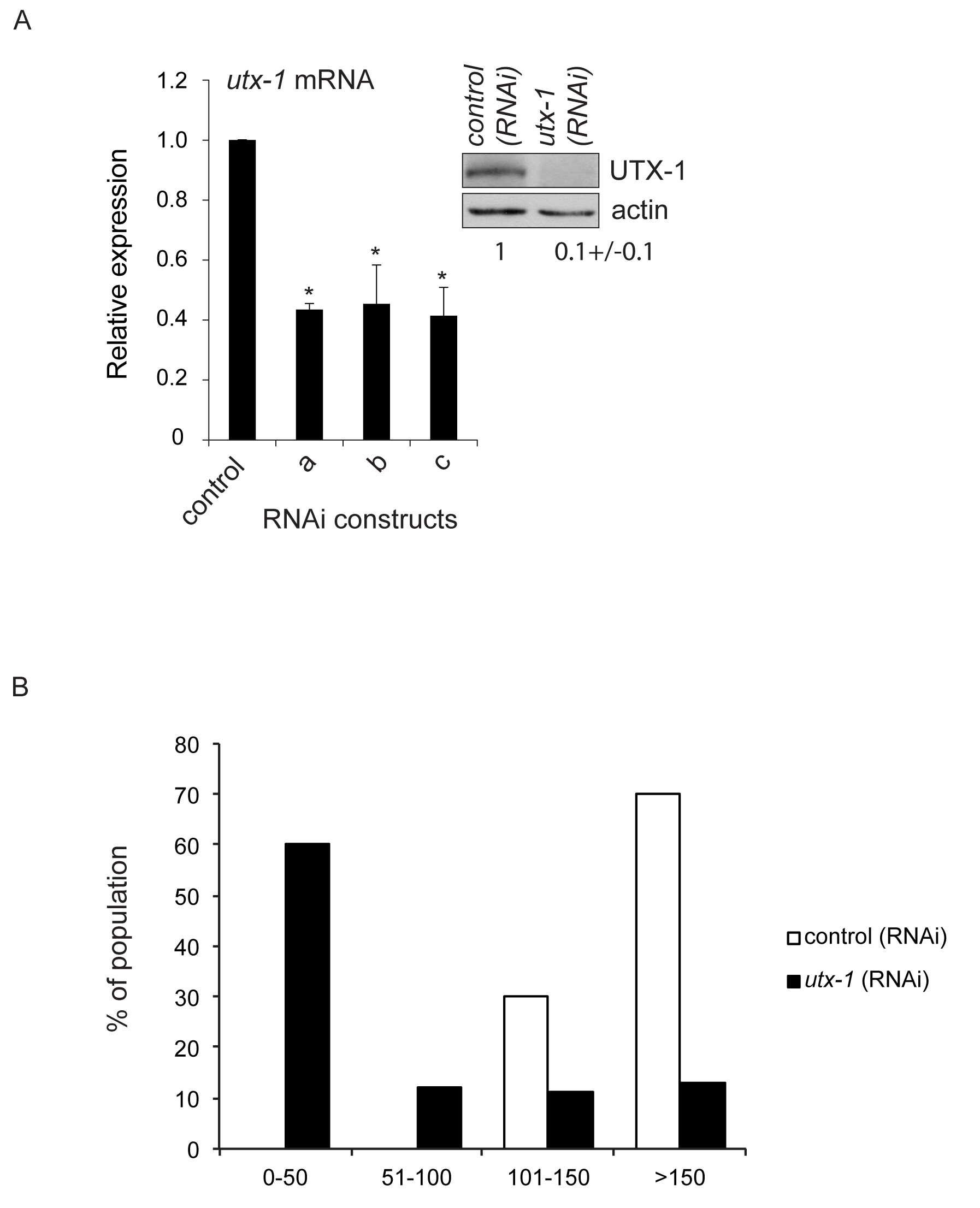

Supplement: Figure S7 — Efficiency of RNAi and brood size in utx-1(RNAi) treated worms. (A) Relative expression of utx-1 mRNA after feeding RNAi treatments using three constructs targeting different regions of utx-1, indicated in Figure 1A. Level of UTX-1 protein after utx-1(RNAi) was measured by Western blot, using a specific antibody against UTX-1. Actin was used as loading control. The signals were quantified using ImageJ program and normalized to actin. Values are relative to control RNAi. Asterisks indicate results different at p<0.01 (Student's t-test). (B) Average number of eggs laid by worms treated with control (white bars) or utx-1(RNAi) (black bars). (TIF) [file pgen.1002647.s007.tif]

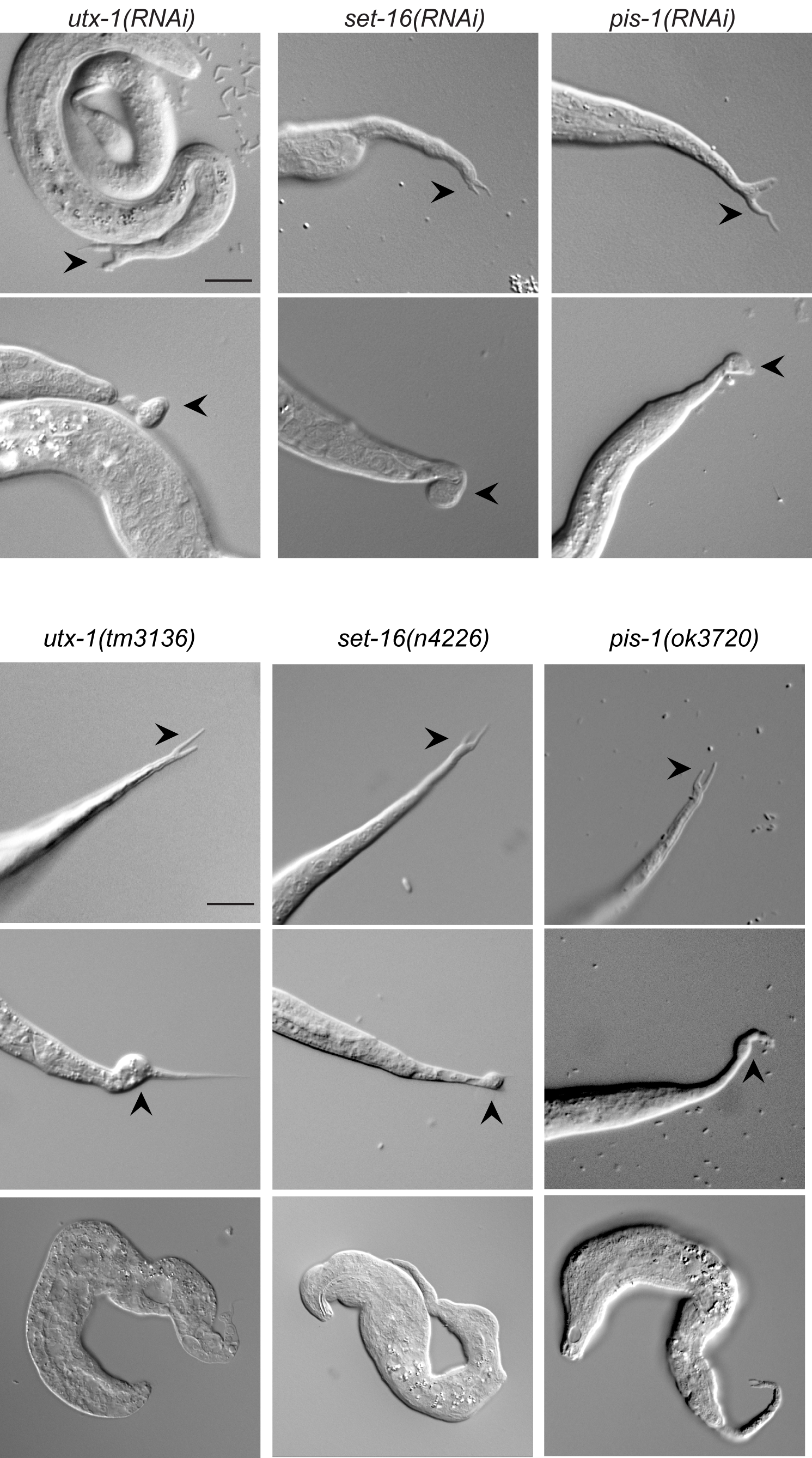

Supplement: Figure S8 — Tail defects associated to loss/downregulation of specific components of the SET-16/UTX-1 complex. Top panels. Representative DIC images of posterior defects observed upon downregulation of utx-1, set-16 and pis-1 using RNAi. F1 L1 are shown. Bottom panels. Representative DIC images of posterior defects observed in utx-1(tm3136), set-16(n4226) and pis-1(ok3720). L1 larvae are shown. Bars are 10 µm. (TIF) [file pgen.1002647.s008.tif]

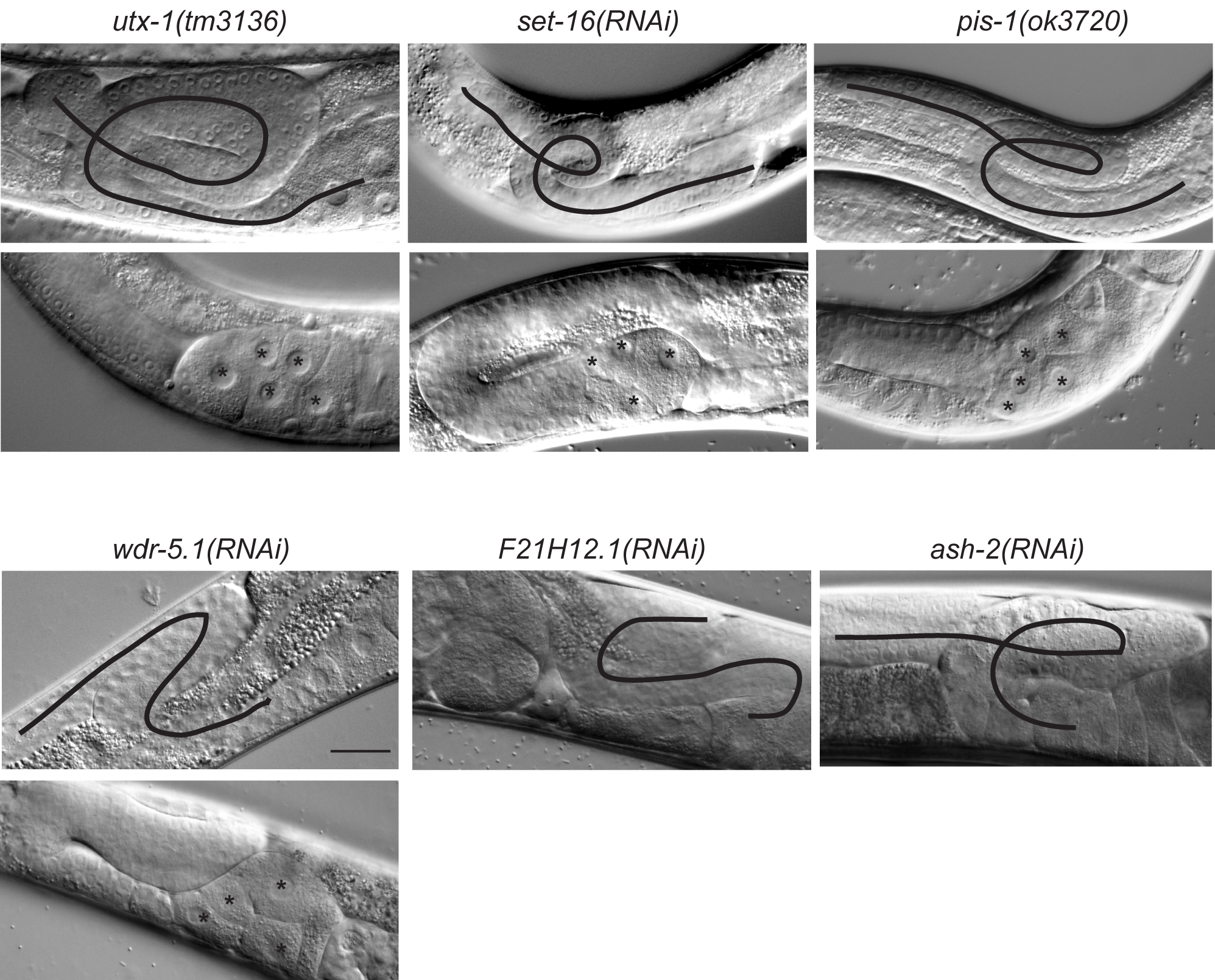

Supplement: Figure S9 — Somatic gonad defects associated to loss/downregulation of the components of SET-16/UTX-1 complex. Representative DIC images of gonadal defects (top: aberrant migration, bottom: oocyte accumulation) observed in utx-1(tm3136), pis-1(ok3720) and after RNAi of the indicated genes. set-16(n4226) dies before gonad migration. Asterisks indicate oocytes accumulation at the distal region of the gonad, black lines indicate the aberrant gonadal migration. Accumulation of oocytes is not observed after down-regulation of ash-2 and wdr-5. In RNAi, F1 or F2 young adult were scored. (TIF) [file pgen.1002647.s009.tif]

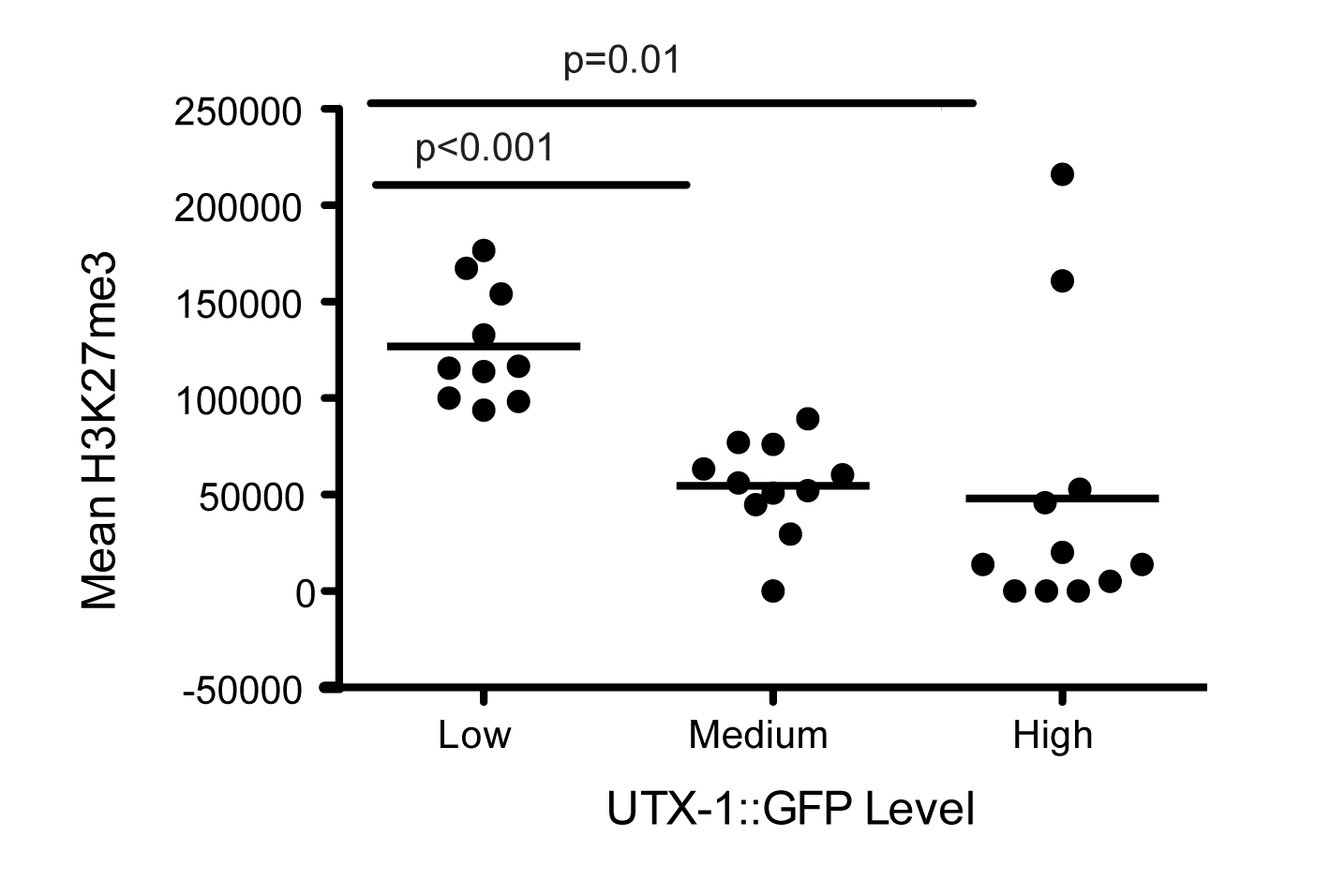

Supplement: Figure S10 — The overexpression level of UTX-1 inversely correlates with H3K27me3 intensity. Level of H3K27me3 in N2 overexpressing wild-type UTX-1::GFP. H3K27me3 and GFP intensities in single intestinal cells (shown in Figure 3B) were analyzed as described in Materials and Methods. Scatter plot presentation of H3K27me3 levels in cells having low (n = 10, range: 0–14807 arbitrary units) medium (n = 11, range: 28645–145977 arbitrary units) or high (n = 11, range: 148852–258798 arbitrary units) levels of UTX-1::GFP. Statistical significance levels are provided. Worms analyzed were from two independent experiments. Mean of H3K27me3 is expressed in arbitrary units. (TIF) [file pgen.1002647.s010.tif]

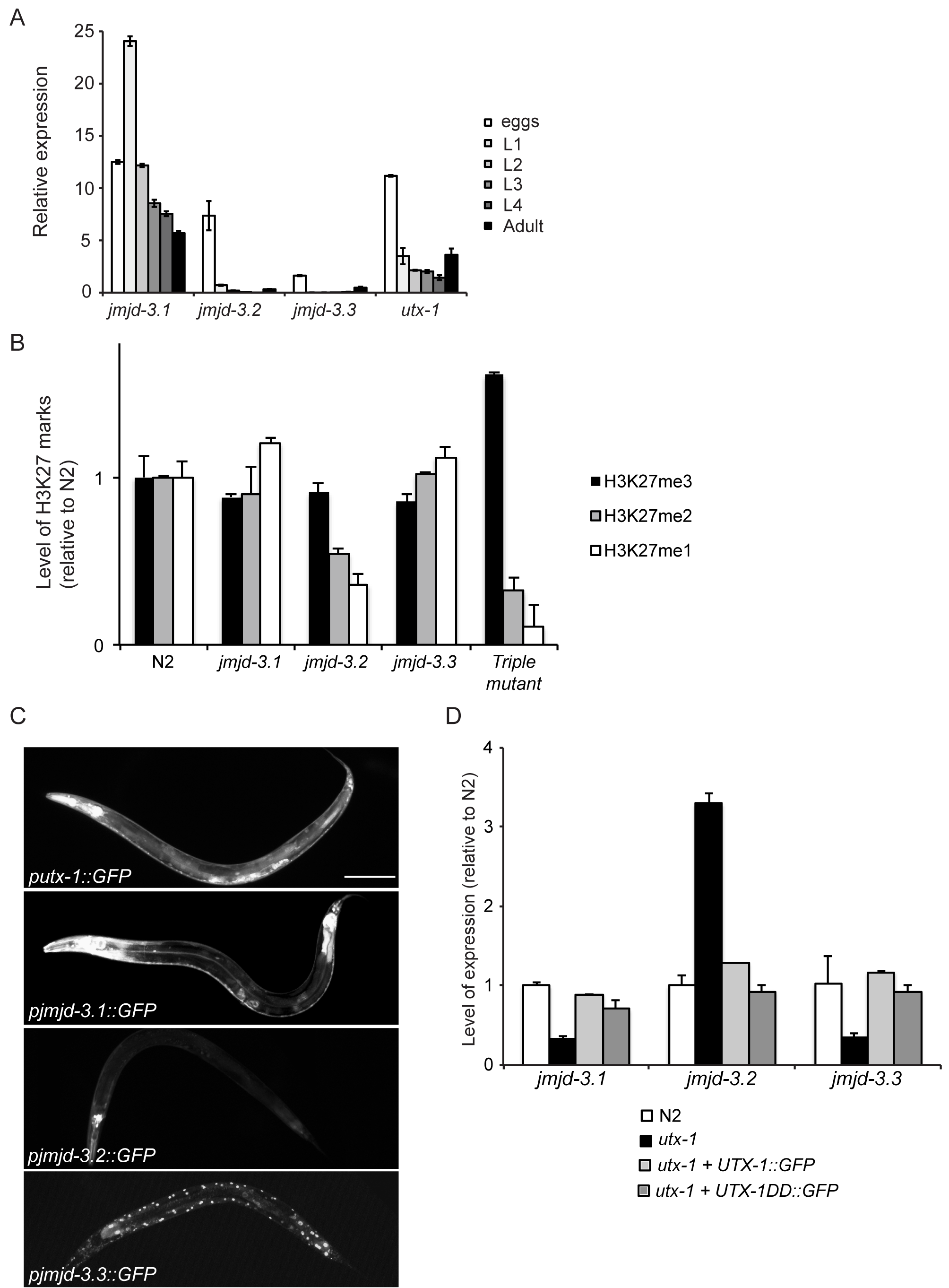

Supplement: Figure S11 — Expression of the KDM6 family members. (A) Relative expression of the KDM6 class members in wild-type animals at different stages, normalized to rpl-26 levels. (B) Quantification of the western blot shown in Figure 4B. Bands were analyzed using ImageJ program and the values reported are relative to N2 levels. Note the increase level of H3K27me3 in the triple mutant. (C) Expression of transcriptional fusion constructs of the four members of the KDM6 family obtained as described in Text S1. Epifluorescence of adult animals is shown. Animals are oriented head to the left, ventral down. Bar is 100 µm. (D) Expression of the JMJD3-like genes in the indicated genetic backgrounds. The levels are relative to N2 and normalized to rpl-26. (TIF) [file pgen.1002647.s011.tif]

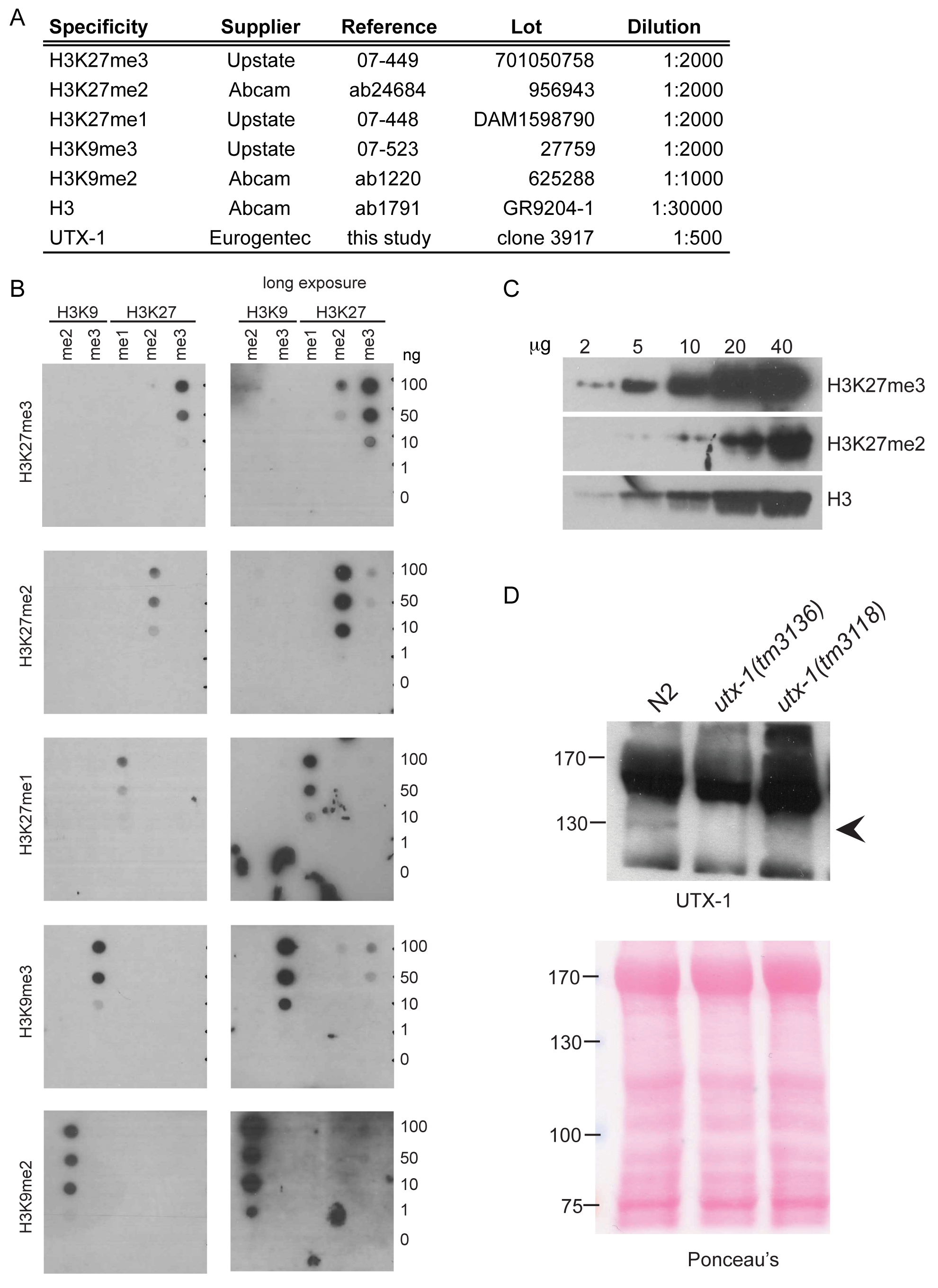

Supplement: Figure S12 — Specificity of the antibodies. A) List of the antibodies used in this study. Specificity, reference, lot number and working dilution of the antibodies are indicated. (B) Specifity of the antibodies used in this study. Dot blots were performed as indicated in Text S1 and Methods and using the following peptides (indicated on the top): Histone H3 di-methyl K9 (H3K9me2), Histone H3 tri-methyl K9 (H3K9me3), Histone H3 mono-methyl K27 (H3K27me1), Histone H3 di-methyl K27 (H3K27me2), Histone H3 tri-methyl K27 (H3K27me3). Different amounts of peptides, ranging from 100 to 1 ng, as indicated on the right side of the blots, were spotted on the membrane before probing with the indicated antibodies (on the left). Two exposures of the same membrane are shown. (C) Efficiency of the antibodies used in this study on worm samples. Different amounts of worm total protein extracts (from 1 to 40 µg) were resolved on a 15% polyacrylamide gel and probed with the indicated antibodies. The western blots indicate that our analysis was performed using the linear range of the antibodies. (D) Specificity of the UTX-1 antibody. Total protein extracts (50 µg) from N2 and utx-1 mutants were analyzed by Western blot using UTX-1 polyclonal antibody. Ponceau's staining of the membrane shows equal loading. Arrowhead indicates the UTX-1 band. (TIF) [file pgen.1002647.s012.tif]
