# Supplementary material for: The C. elegans H3K27 Demethylase UTX-1 Is Essential for Normal Development, Independent of Its Enzymatic Activity
Source: PLoS Genet. 2012 May 3;8(5):e1002647. doi: 10.1371/journal.pgen.1002647 (PMC3342935; doi:10.1371/journal.pgen.1002647)
Supplement: Text S1 — Supporting Materials and Methods. (DOCX) [file pgen.1002647.s015.docx]

**Text S1**

***Reporter construction***

To generate transcriptional reporters for *utx-1, jmjd-3.1, jmjd-3.2 and jmjd-3.3* we used the PCR fusion based approach [1]. The fused PCR products were derived from two overlapping PCR fragments. One contained the promoter region and the first exon of each gene*,* the other contained *gfp* and the *unc-54* 3′UTR amplified from pPD95.75 (Fire lab). The resulting PCR products were co-injected with ttx-3p::RFP fluorescence marker into wild type animals and at least two independent transgenic lines were obtained for each construct. All primer sequences are available upon request.

***Dot Blot***

Synthetic biotinylated peptides of histone H3 lysine 27 mono-, di- and tri-methylated (H3K27me1, H3K27me2 and H3K27me3) and histone H3 lysine 9 di- and tri-methylated (H3K9me2 and H3K9me3) (Jerini Peptide Technologies) were spotted in dilution series (ranging from 100 to 1 ng) on Nitrocellulose membrane (Hybond-C Extra; Amersham Biosciences). The membrane was blocked for 1 hour in 5% non-fat milk dissolved in PBS-T (PBS (pH 7.2), 0.01% Tween-20) and the following antibodies were tested for specificity at the indicated concentrations: H3K27me1 (Upstate, 07-448, 1:2000); H3K27me2 (Abcam, ab24684, 1:2000), H3K27me3 (Upstate, 07-449, 1:1:2000), H3K9me2 (Abcam, ab1220, 1:1000) and H3K9me3 (Upstate, 07-523, 1:2000). After incubation with primary antibodies, the membrane was washed four times and probed with secondary anti-rabbit or anti-mouse HRP antibodies (Vector laboratories, dilution 1:10,000). After 3 washes in PBS-T, the signal was detected by enhanced chemiluminiscence (SuperSignal West Pico Chemiluminescent Substrate, Thermo scientific). Blots were exposed to films for different time exposures.

***Reference for Supplementary Materials and Methods***

1. Hobert O (2002) PCR fusion-based approach to create reporter gene constructs for expression analysis in transgenic C. elegans. BioTechniques 32: 728-730.
